# Dual Ferro‐/Piezo‐Electric Coupling in Two‐Dimensional [Bi_2_O_2_]‐Based Layered Structures for Synergistic Harvesting of Mechanical and Solar Energy

**DOI:** 10.1002/EXP.20250105

**Published:** 2026-05-08

**Authors:** Daotong You, Xingwang Long, Zhiyong Yang, Lei Liu, Jianbang Chen, Tuan Guo

**Affiliations:** ^1^ Institute of Photonics Technology, College of Physics and Optoelectronic Engineering Jinan University Guangzhou P. R. China; ^2^ School of Optoelectronic Engineering Guangxi Key Laboratory of Optoelectronic Information Processing, Guilin University of Electronic Technology Guilin P. R. China

**Keywords:** [Bi_2_O_2_]‐based layered structure, dual‐piezoelectric effect, inter‐plane 2D/2D heterojunction

## Abstract

Piezoelectric‐assisted photocatalytic systems, synergistically harnessing mechanical and solar energy, represent a promising frontier for energy conversion and environmental remediation. However, their practical implementation remains challenged by interfacial screening effects and inefficient carrier dynamics in conventional heterojunctions. Herein, we fabricated an inter‐plane 2D/2D heterojunction of polar [Bi_2_O_2_]‐based layered compounds (BiOBr@Bi_5_Ti_3_FeO_15_) featuring matched electronic structures and dual piezoelectric response. This excellent structure facilitates the formation of interfacial chemical bonds (Bi‐O‐Ti and Bi‐O‐Fe bonds) and strong electronic interactions, which synergistically enhance piezoelectric and photoelectric properties by acting as charge transfer channels and strain‐concentrated centers. Furthermore, under combined light illumination and ultrasonic vibration, a dual piezoelectric polarization field is established, which alternately breaks interfacial shielding effects while modulating interfacial band bending to achieve a Z‐scheme charge transfer mechanism. This mechanism promotes photogenerated charge migration and redox kinetics, ultimately enabling full utilization of solar and mechanical energy. Consequently, the optimized BiOBr@Bi_5_Ti_3_FeO_15_ achieved complete (100%) piezo‐photocatalytic degradation of RhB (25 mg L^−1^) within 6 min, exhibiting a degradation rate of 0.5399 min^−1^, 1.76‐fold and 128.5‐fold higher than standalone photocatalysis (0.3057 min^−1^) and piezocatalysis (0.0042 min^−1^), respectively. This work provides a novel strategy for designing atomic‐level 2D/2D ferro‐/piezoelectric heterojunctions with tailored interfacial structures, effectively addressing the screening effect and weak interfacial interactions inherent to conventional piezoelectric heterojunctions, while advancing applications in energy and environmental technologies.

## Graphical abstract

Inter‐plane 2D/2D nanosheet heterojunction based on two piezoelectric [Bi_2_O_2_]‐based layered compounds is constructed. Significantly increased piezoelectric properties of BiOBr@Bi_5_Ti_3_FeO_15_ by morphological regulation and interface chemical bonds. Dual‐piezoelectric field can modulate interfacial electric field and the interfacial band bending to facilitate charge transfer.

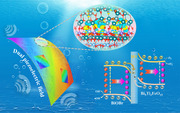

## 1 Introduction

The construction of built‐in electric fields at semiconductor interfaces has been demonstrated to be effective in driving photogenerated carrier separation during photocatalysis, playing a crucial role in promoting environment remediation and energy conversion [1, 2, 3]. In this context, the piezoelectric potential generated by ferro‐/piezoelectric materials under mechanical stimulation represents an emerging strategy for enhancing built‐in electric fields, which in turn manipulates band structure and facilitates photogenerated carrier separation [4, 5]. Moreover, piezo‐photocatalysis synergistically combines the complementary advantages of two catalytic approaches: (mild conditions, low energy consumption, and strong oxidation) and piezo‐catalysis (environmental protection, low cost, and high efficiency) [6].

Currently, various piezoelectric materials including BaTiO_3_, ZnO, KNbO_3_, NaNbO_3_, MoS_2_, and BiFeO_3_ have been explored as potential piezo‐photocatalysts [7, 8, 9, 10, 11]. Among them, the lead‐free ferro‐/piezo‐electric materials Bi_5_Ti_3_FeO_15_ features a typical bismuth‐based layered structure comprising alternating fluorite [Bi_2_O_2_]^2+^ layer and the perovskite [Bi_3_Ti_3_FeO_13_]^2−^ layer, which endow it with inherent ferro‐/piezo‐electric properties [12]. Moreover, Bi_5_Ti_3_FeO_15_ demonstrates a narrow bandgap (≈2.08–2.38 eV), positioning it as a promising candidate for piezo‐photocatalytic applications [13]. Nevertheless, its practical implementation faces three primary limitations: (1) weak piezoelectric response for mechanical energy, (2) insufficient catalytic active sites, and (3) rapid recombination of photogenerated carriers, which collectively constrain its piezo‐photocatalytic efficiency [14, 15]. Heterojunction engineering has emerged as a promising strategy to address these challenges by synergistically coupling the intrinsic piezoelectric polarization field with interfacial electric fields at heterojunction boundaries [16, 17]. Several Bi_5_Ti_3_FeO_15_‐based piezoelectric heterojunctions, such as Bi_5_Ti_3_FeO_15_/g‐C_3_N_4_ and Bi_5_Ti_3_FeO_15_/BiOCl, have demonstrated enhanced piezo‐photocatalytic performance [18, 19]. However, a critical limitation persists in systems incorporating non‐piezoelectric semiconductors. These components induce a shielding effect that impedes the efficient separation of space charges and severely restricts interfacial charge transfer between the piezoelectric and non‐piezoelectric components [20]. Such shielding effect not only diminishes the piezoelectric polarization but also weakens the overall catalytic performance. Additionally, lattice mismatch at heterojunction interfaces frequently generates defect states and elevates interfacial resistance, creating energy barriers that suppress carrier mobility and diminish piezo‐photocatalytic efficiency [21].

Consequently, developing dual‐piezoelectric heterojunctions with matched electronic structures and dual piezoelectric effects is a worthwhile attempt to overcome lattice mismatch and alternately break the interfacial shielding effects [22, 23]. Although previous studies have demonstrated the potential of combining conventional piezoelectric materials like ZnO/MoS_2_ (exhibiting 2.4 times photocurrent enhancement over ZnO) and NaNbO_3_/g‐C_3_N_4_ (demonstrating 87.3% antibiotic degradation within 10 min) [24, 25], these systems remain fundamentally constrained by interfacial defects arising from structural dissimilarities between components. As a result, exploiting an appropriate dual piezoelectric material is still a meaningful research topic. Notably, both BiOBr and Bi_5_Ti_3_FeO_15_ belong to the [Bi_2_O_2_]^2+^‐based layered family, sharing structural homology through alternating [Bi_2_O_2_]^2+^ slabs and X_2_^−^ layers (X = Br^−^ for BiOBr, [Bi_3_Ti_3_FeO_13_]^2−^ for Bi_5_Ti_3_FeO_15_) [26]. This atomic‐level compatibility ensures synergistic functionality integration: piezoelectric BiOBr generates dynamic polarization under stress, while ferroelectric Bi_5_Ti_3_FeO_15_ maintains permanent dipole alignment. The combination of their structural congruence and type‐II band alignment establishes a compelling foundation for constructing a Bi_5_Ti_3_FeO_15_/BiOBr dual‐piezoelectric heterostructure, potentially overcoming the limitations observed in conventional composite systems.

Beyond the inherent crystal structure and band configuration of heterojunctions, several critical factors significantly influence the optimization of piezo‐photocatalytic activities. These include morphological regulation, polar direction sites, synergistic pressure response, and active site availability [27, 28]. Conventional nanoparticle‐based piezoelectric heterojunctions frequently suffer from limited contact and inadequate interface dynamics, leading to random dipole arrangement and consequently unsatisfactory piezoelectric properties [29]. To address these limitations, precise control over the orientation and arrangement of dipole moments in piezoelectric heterojunctions has become imperative in heterojunction design. The emerging paradigm of 2D/2D ferroelectric/piezoelectric heterostructures presents transformative advantages in this context. These structures exhibit enhanced interface contact through their unique face‐to‐face configuration, surpassing traditional heterojunction systems [30]. Compared to irregular nanoparticles and bulk materials, 2D heterostructures demonstrate dual enhancement mechanisms for piezo‐photocatalysis: First, their atomic‐scale thickness and exceptional flexibility enable greater deformation under mechanical forces, generating substantially larger piezopotentials and stronger piezoelectric fields [31]. Second, the 2D architecture provides directional charge transport channels, reducing the distance photogenerated carriers travel from the interior to the surface. Furthermore, these materials expose numerous surface unsaturated atoms that serve as active sites, significantly improving the adsorption and transformation of reactant molecules [32].

Based on the above considerations, we propose an innovative approach that strategically combines BiOBr with Bi_5_Ti_3_FeO_15_, leveraging their similar [Bi_2_O_2_]^2+^ layer structures and dual piezoelectric properties to design an inter‐plane 2D/2D nanosheet ferro‐/piezo‐electric heterojunction. This configuration features enhanced interfacial contact and synergistic effects, thereby inducing polar interactions and amplifying charge transfer. The innovative design of BiOBr@Bi_5_Ti_3_FeO_15_ was validated through comprehensive COMSOL simulations and piezoresponse force microscopy (PFM) analyses, demonstrating superior piezoelectric performance compared to individual components. The heterojunction structure can generate dual piezoelectric polarization under the combined action of light irradiation and ultrasonic vibration, effectively modulating the interfacial electric field and band bending. This enables a direct Z‐scheme charge transfer mechanism, ultimately achieving exceptional piezo‐photocatalytic performance for organic pollutant elimination. Specifically, the BiOBr@Bi_5_Ti_3_FeO_15_ demonstrated a degradation rate of 0.5399 min^−1^ for high‐concentration RhB, which was 3.23 times and 7.95 times higher than those of individual BiOBr (0.1672 min^−1^) and Bi_5_Ti_3_FeO_15_ (0.0679 min^−1^), respectively. In conclusion, the construction of inter‐plane 2D/2D heterojunction materials with dual piezo‐responsive and the systematic revelation of their related mechanisms not only contribute to fundamental understanding of piezoelectric and optoelectronic properties, but also provide a valuable framework for developing high‐performance piezo‐photocatalytic systems.

## 2 Experimental Section

### 2.1 Synthesis of Thin Square Bi_5_Ti_3_FeO_15_ Nanosheets

**FIGURE 1:**
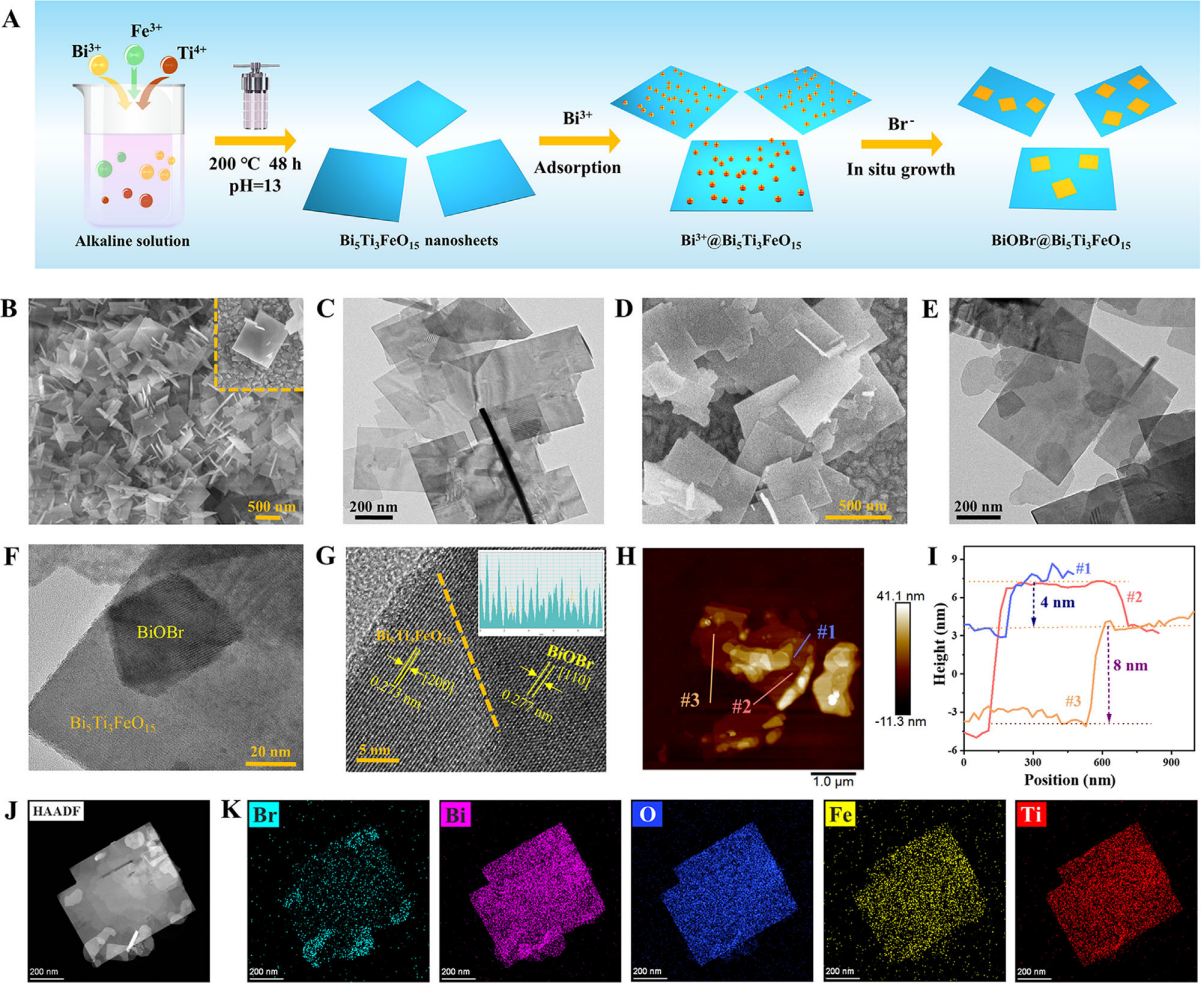
(A) Schematic illustration of the in situ synthetic process of 2D/2D BOB@BTFO nanosheets heterojunction. (B) SEM image and (C) TEM image of BTFO nanosheets. (D) SEM image, (E) TEM image, (F,G) HRTEM image (inset of atomic intensity profile along the dashed line at the interface), (H) AFM image, and (I) corresponding height distribution of BOB@BTFO nanosheets heterojunction. (J) HAADF‐STEM image of BOB@BTFO nanosheets heterojunction and (K) corresponding EDX mapping for Br, Bi, O, Fe, and Ti elements.

Bi_5_Ti_3_FeO_15_ (BTFO) nanosheets were synthesized via a hydrothermal approach (Figure 1A). Initially, 5 mmol of Bi(NO_3_)_3_ **·**5H_2_O was dissolved in 20 mL of deionized water under continuous stirring for 1 h. Then, the solution was then combined with 3 mmol of tetrabutyl titanate and 1 mmol of Fe(NO_3_)_3_ **·**9H_2_O, followed by 20 min of additional stirring. Subsequently, a 2 mol/L NaOH aqueous solution was gradually added to adjust the pH to 13, with continuous agitation maintained for 2 h. The obtained yellow solution was transferred to a 100 mL polytetrafluoroethylene‐lined autoclave, sealed, and subjected to hydrothermal treatment at 200°C for 48 h. Finally, the BTFO nanosheets were collected through centrifugation and washed three times with deionized water.

### 2.2 In Situ Construction of Inter‐Plane 2D/2D BiOBr@Bi_5_Ti_3_FeO_15_ Heterojunction

The BiOBr (BOB) phase was epitaxially grown on BTFO nanosheets via a chemical precipitation method (Figure 1A). Initially, 2 mmol of as‐synthesized BTFO nanosheets were added to 40 mL of deionized water and stirred for 30 min. Subsequently, 1 mmol Bi(NO_3_)_3_·5H_2_O was introduced into the aforementioned solutions and magnetically stirred for 1 h. A stoichiometric KBr solution (1 mmol in 30 mL deionized water) was then added dropwise, followed by 1 h of continuous stirring. After standing for 2 h, the precipitate was collected by centrifugation, washed alternately with deionized water and ethanol five times, and dried at 60°C for 12 h to obtain BOB@BTFO heterojunctions. By varying the molar ratio of BOB and BTFO, different ratios of BOB@BTFO heterojunctions (ratios 1:1, 1:2, and 1:3) were synthesized. For comparison, pristine BOB nanosheets were synthesized under the same synthesis without BTFO addition.

### 2.3 Characterization

Detailed descriptions of characterization methods and instruments were provided in the Supporting Information.

### 2.4 Theoretical Calculation

#### 2.4.1 Piezoelectric Potential Simulation

The piezoelectric potential distribution under ultrasonic vibration‐induced mechanical stress was simulated using the piezoelectric module of COMSOL Multiphysics 5.4. The geometric dimensions of BiOBr (BOB) nanosheets were set as 150 nm × 150 nm × 4 nm, while Bi_5_Ti_3_FeO_15_ (BTFO) nanosheets were modeled as 500 nm × 500 nm × 8 nm to match experimental morphologies. A uniform vertical compressive stress of 100 MPa was applied to the surfaces of individual BOB, BTFO, and BOB@BTFO heterostructure, to accurately replicate practical mechanical loading conditions. Material‐specific parameters including elastic compliance matrix, piezoelectric strain coefficients, and dielectric permittivity were incorporated into the finite element model based on experimentally characterized values. Through iterative boundary condition optimization and mesh convergence verification, the spatial distribution of surface piezoelectric potential was numerically resolved, providing critical insights into the stress‐polarization coupling behavior at nanoscale interfaces.

#### 2.4.2 Theoretical Simulation of Density Functional Theory (DFT)

All DFT calculations were performed using Quantum Espresso (QE). The Perdew–Burke–Ernzerhof (PBE) exchange‐correlation functional and projector augmented wave (PAW) pseudopotential were adopted with spin‐polarization. During structure optimization, the total energy convergence criterion was set to 10^−6^ eV, and the atoms were relaxed until the maximum force on any atom fell below 0.01 eV Å^−1^. Gaussian smearing of 0.05 eV was applied to orbital occupations. A plane‐wave cutoff energy of 480 eV was utilized throughout all computations. Brillouin‐zone integrations were performed using Monkhorst–Pack (MP) grids of special points with the separation of 0.08 Å^−1^. The optimized lattice parameters were determined as: BOB: *a* = *b* = 3.93 Å, *c* = 8.39 Å. BTFO: *a* = *b* = 5.47 Å, *c* = 41.25 Å. For BOB, the (110) crystallographic plane was selected and expanded into a 2 × 1 supercell configuration, yielding surface dimensions of *u* = 16.79 Å and *v* = 5.55 Å. In the case of BTFO, surface construction employed the elongated (002) basal plane along the c‐axis direction, with a 3 × 1 supercell expansion producing surface parameters *u* = 16.40 Å and *v* = 5.44 Å. The average in‐plane lattice parameters (*u* = 16.59 Å, *v* = 5.49 Å) were selected as the interfacial matching basis for heterojunction construction. The optimized heterostructure model incorporated a 15 Å vacuum layer along the *z*‐axis to eliminate spurious periodic interactions.

### 2.5 Catalytic Performance

The piezo‐photocatalytic degradation performance of BOB, BTFO, and BOB@BTFO was evaluated using rhodamine B (RhB, 25 mg L^−1^) as the model pollutant. In each test, 50 mg catalyst was dispersed in 100 mL RhB solution, followed by 15 min dark adsorption to achieve adsorption–desorption equilibrium. The degradation process was initiated under simultaneous ultrasonic irradiation (100 W, 40 kHz) and xenon lamp illumination (300 W, AM 1.5G spectrum). Reaction aliquots (1.5 mL) were collected at 2‐min intervals, immediately centrifuged to remove catalyst particles, and analyzed by UV–vis spectrophotometry (*λ* = 554 nm) to determine residual RhB concentration. Meanwhile, the piezo‐photocatalytic degradation activity of BOB@BTFO for chloramphenicol (CTC), tetracycline (TC), and ofloxacin (OFLO) were investigated.

## 3 Results and Discussions

### 3.1 Design and Morphology of 2D/2D [Bi_2_O_2_]‐Based Layered Structures Heterojunction

As shown in Figure 1B,C, the pure BTFO demonstrated a well‐defined 2D nanosheet morphology characterized by smooth surface and a size ranging from 400 to 500 nm. The high‐resolution transmission electron microscope (HRTEM) image of the BTFO nanosheets (Figure S1) revealed a lattice spacing of 0.273 nm, corresponding to the (200) plane of BTFO [21]. The scanning electron microscope (SEM) images revealed that pure BOB formed aggregated nanosheet structure (Figure S2). SEM and TEM characterization (Figure 1D,E) confirmed the successful construction of 2D/2D heterostructures, where 100–200 nm BOB nanosheets were uniformly grown on larger BTFO nanosheets, forming intimate interfacial contacts. HRTEM images (Figure 1F,G) clearly revealed the coherent interface between components, with lattice spacings of 0.277 and 0.273 nm matching the BOB (110) planes and the BTFO (200) planes, respectively. The partial peak intensities of lattice fringes at the interface between BOB and BTFO were significantly lower than other peaks (inset of Figure 1G), which not only indicates defect formation but also demonstrates that the interfacial morphology is predominantly governed by lattice distortion rather than interlayer stacking [33]. The thicknesses of BOB and BTFO were determined by atomic force microscopy (AFM) characterization of approximately 4 and 8 nm, respectively (Figure 1H,I). High‐angle annular dark field scanning transmission electron microscopy (HAADF‐STEM) (Figure 1J) revealed distinct layer contrast between the upper BOB (lighter contrast) and underlying BTFO (darker contrast), confirming the 2D/2D heterostructure. Additionally, energy dispersive X‐ray spectroscopy (EDX) verified the composition and distribution of elementals within the BOB@BTFO (Figure 1K and Figure S3), clearly distinguishing the spectra and corresponding content of Br, Bi, O, Fe, and Ti. Collectively, these analyses confirm the successful synthesis of ultrathin 2D/2D BOB@BTFO heterojunctions.

### 3.2 Crystal Structure and Interface Chemical Bonds of 2D/2D Heterojunction

**FIGURE 2:**
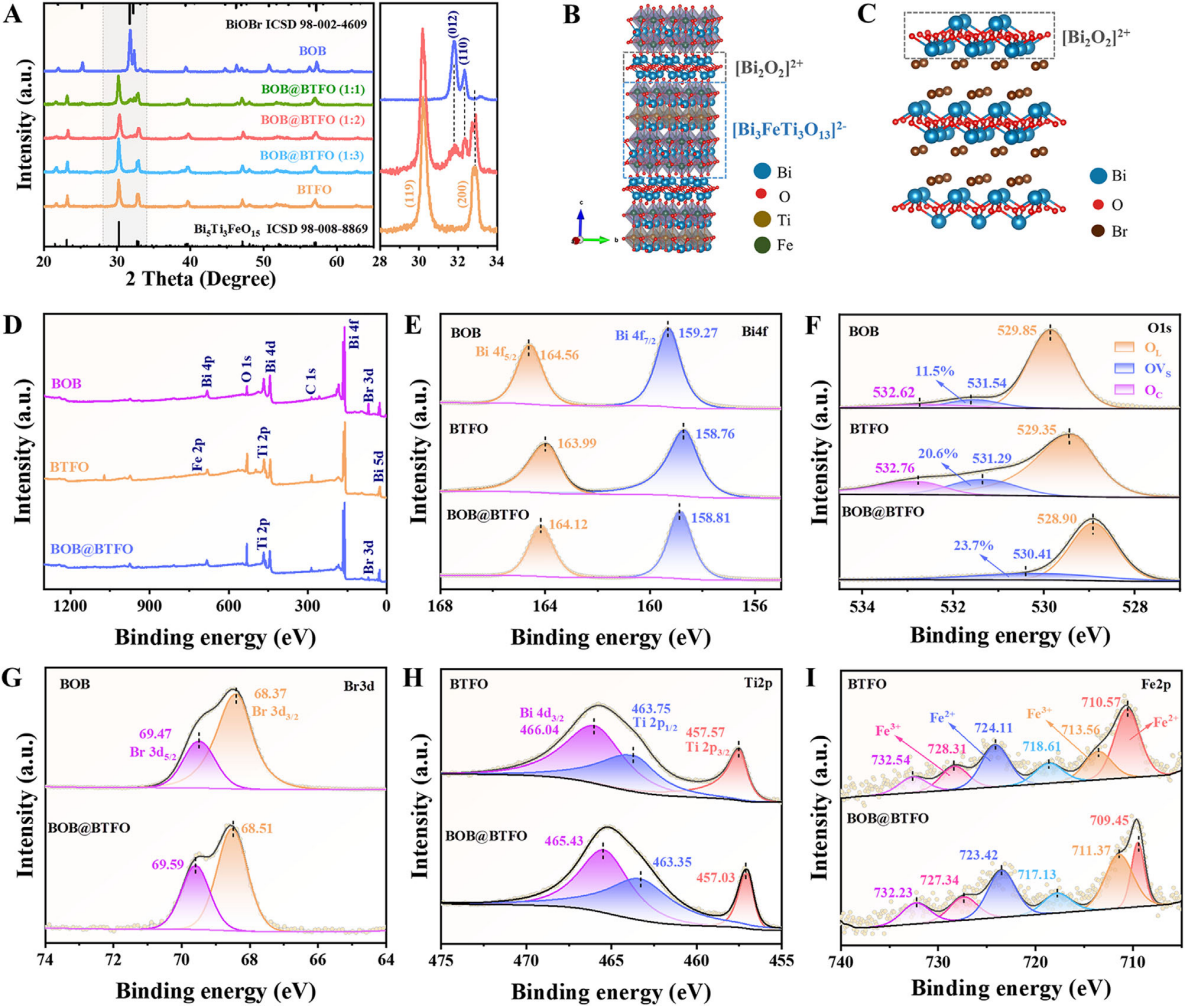
(A) XRD patterns of BOB, BTFO, and BOB@BTFO, the crystal structure models of (B) BTFO and (C) BOB, (D) XPS full spectra and high‐resolution XPS spectra of BOB, BTFO, and BOB@BTFO: (E) Bi 4f, (F) O 1s, (G) Br 3d, (H) Ti 2p, (I) Fe 2p.

As illustrated in Figure 2A, the X‐ray diffraction (XRD) patterns of BTFO were well‐indexed to the orthorhombic phase (ICSD 98‐008‐8869) [12], confirming its structural purity. The crystal structure of BTFO (Figure 2B) features a sandwich‐like arrangement of alternating fluorite‐type [Bi_2_O_2_]^2^^+^ layers and perovskite‐like [Bi_3_FeTi_3_O_13_]^2^^−^ slabs. Within the [Bi_3_FeTi_3_O_13_]^2^^−^ layers, Bi^3^^+^ ions occupy distorted octahedral sites owing to the stereochemical activity of their 6s^2^ lone pairs, while Bi^3^^+^ in the [Bi_2_O_2_]^2^^+^ layers exhibit a distorted tetrahedral coordination with four oxygen anions [34]. In contrast, BOB crystallizes in a tetragonal Sillén structure (ICSD 98‐002‐4609) [35], characterized by alternating [Bi_2_O_2_]^2^^+^ layers and dual Br^−^ sheets (Figure 2C). The strong ionic bonding within the [Bi_2_O_2_]^2^^+^‐Br^−^ layers, coupled with weak van der Waals interactions between adjacent Br^−^ sheets, generates a pronounced intrinsic dipole moment, endowing BOB with exceptional piezoelectricity [35]. For the BOB@BTFO heterojunction, the XRD pattern retained all characteristic peaks of BTFO, with additional peaks at 31.7° and 32.2° corresponding to the (012) and (110) planes of BOB (Figure 2A). Notably, the intensity of these diffraction peaks increased with the loading amount of BOB. Further structural validation was provided by Raman spectroscopy (Figure S4), where the peak at 161 cm^−1^ in BOB@BTFO aligns with the vibrational mode of BOB [36].

The elemental composition and chemical states of the samples were further analyzed by X‐ray photoelectron spectroscopy (XPS). Survey spectra (Figure 2D) confirmed the presence of Bi, Br, and O in BOB, Bi, Ti, Fe, and O in BTFO, and main five elements (Bi, Br, Ti, Fe, and O) in BOB@BTFO. High‐resolution Bi 4f XPS spectra (Figure 2E) revealed two peaks at 164.12 eV (Bi 4f_5/2_) and 158.81 eV (Bi 4f_7/2_) for BOB@BTFO, exhibiting a positive shift relative to BTFO (163.99 and 158.76 eV) but a negative shift compared to BOB (164.56 and 159.27 eV), indicating electron redistribution at the Bi^3^^+^ [10, 11, 37]. The O 1s XPS spectrum of BOB@BTFO (Figure 2F) exhibited two distinct peaks at 530.41 eV (oxygen vacancies, OVs) and 528.90 eV (lattice oxygen, O_L_), with notable binding energy shifts observed compared to BOB and BTFO. Quantitative analysis revealed that the OVs concentrations were calculated to be 11.5%, 20.6%, and 23.7% for BOB, BTFO, and BOB@BTFO, respectively. Electron paramagnetic resonance (EPR) measurements revealed characteristic oxygen vacancy signals at *g* = 2.002, with relative intensities progression: BOB@BTFO > BTFO > BOB (Figure S5), consistent with XPS quantification. This interfacial oxygen vacancy enrichment provides direct evidence for lattice distortion‐induced bond reconfiguration at heterojunction boundaries, which modifies oxygen coordination geometry and redistributes electron density around oxygen species [38]. Notably, Br 3d XPS peaks in BOB@BTFO shifted positively (69.59 eV for Br 3d_5/2_; 68.51 eV for Br 3d_3/2_) relative to BOB (Figure 2G), while Ti 2p XPS peaks in BTFO (457.57 eV for Ti 2p_3/2_; 463.75 eV for Ti 2p_1/2)_ shifted negatively to 457.03 and 463.35 eV in BOB@BTFO (Figure 2H) [27, 28]. Concurrently, Fe 2p XPS spectra (Figure 2I) exhibited dual oxidation states (Fe^2^^+^: 709.45 eV/723.42 eV; Fe^3^^+^: 711.37 eV/727.34 eV), with peak shifts indicating electron transfer from BOB to BTFO [10, 11, 38]. The XPS results clearly demonstrate the BOB@BTFO with enhanced chemical bonding is successfully constructed, rather than a simple physical mixture.

### 3.3 Ferro‐/Piezo‐Electric Analysis and Simulated Piezopotentials Under Applied Mechanical Energy

**FIGURE 3:**
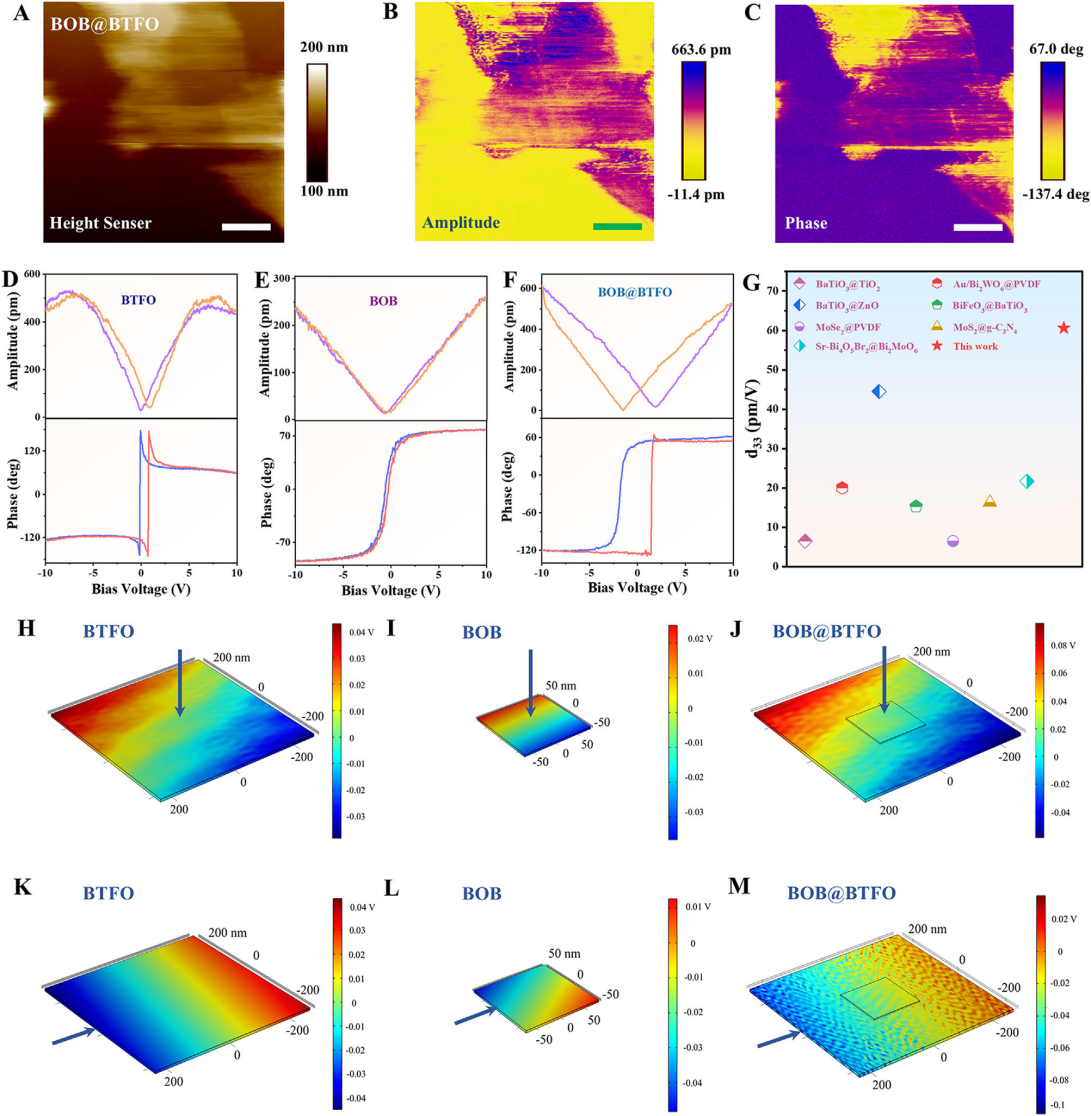
(A) Surface morphology, (B) amplitude image and (C) phase image of BOB@BTFO samples, the scale size was 500 nm. Piezoelectric amplitude‐voltage curves and piezoelectric phase‐voltage curves for (D) BOB, (E) BTFO, and (F) BOB@BTFO. (G) comparison data of the piezoelectric coefficient (d_33_) of BOB@BTFO with reported piezoelectric material. Simulated piezo‐potential distributions for (H, K) BTFO, (I, L) BOB, and (J, M) BOB@BTFO under pressure in the vertical and parallel direction.

The nanosheet morphology of BOB@BTFO heterostructures is evident in Figure 3A. The amplitude and phase responses of the materials were measured by applying a 3 V voltage. As shown in Figure 3B,C, the phase and amplitude distribution of BOB@BTFO displayed superior uniformity in phase distribution and high amplitude contrast. Piezoresponse force microscopy (PFM) tests yielded characteristic amplitude–voltage and phase–voltage curves. The displacement field loops directly demonstrated nanoscale ferroelectric domain responses to electric fields, confirming piezoelectric behavior [39]. As shown in Figure 3D–F, compared to BOB and BTFO, BOB@BTFO exhibited a typical butterfly‐shaped piezoelectric hysteresis loop and approximately 180° phase shift observed at ±10 V, suggesting enhanced piezoelectricity in the heterojunction and switchable ferroelectric polarization at interface/surface regions. The piezoelectric coefficient (*d*_33_) was gained from the PFM amplitude data using the formula (*d*_33_ = *a V* ^−1^, *a*: amplitude, *V*: voltage) [39]. BOB@BTFO demonstrated a superior *d*_33_ of 60.65 pm V^−1^, surpassing both BOB (26.03 pm V^−1^) and BTFO (52.11 pm V^−1^) (Figure S6). Furthermore, the *d*_33_ of our synthesized BOB@BTFO nanosheet heterojunction outperformed state‐of‐the‐art heterostructured piezoelectric materials reported to date, including BaTiO_3_@TiO_2_ (6.5 pm V^−1^), Au@Bi_2_WO_6_@PVDF (20.0 pm V^−1^), BaTiO_3_@ZnO (44.5 pm V^−1^) (Figure 3G and Table S1). These results demonstrate that the [Bi_2_O_2_]^2^^+^‐based 2D/2D heterojunction is beneficial for enhancing piezoelectric performance. The polarization‐electric field (P‐E) loops of BTFO exhibited a distinct hysteresis loop (Figure S7), indicating its excellent ferroelectric properties. While the ferroelectric polarization value of BOB@BTFO was weakened compared to BTFO, this was mainly due to the absence of ferroelectric properties of BOB. Finite element simulations were conducted using COMSOL Multiphysics 5.4. The calculated piezoelectric potential and surface strain distribution for BOB, BTFO, and BOB@BTFO under 100 MPa applied stress along vertical and parallel directions were presented in Figure 3H–J and Figure 3K–M, respectively. The vertical loading direction generated significantly larger piezopotential differences than parallel loading under equivalent stress conditions. It was worth noting that no matter where the pressure comes from, the piezopotential difference in the BOB@BTFO (155.12 mV) was always higher than that in BOB (61.61 mV) and BTFO (80.97 mV). The determination of interfacial lattice mismatch rates based on first‐principles calculations is as follows: Along the *uu*‐direction: 1.2% for BOB [(16.79–16.59)/16.59] and 1.1% for BTFO. Along the *vv*‐direction: 1.1% for BOB and 0.9% for BTFO. The low interfacial lattice mismatch confirms the compatibility of the analogous [Bi_2_O_2_]^2^^+^ layered structures. This results in an interfacial lattice mismatch rate of ≈1.0%, indicating strong interfacial coherence, which facilitates strain transfer across the interface.

### 3.4 Piezo‐Photocatalytic Performance Under Applied Mechanical and Solar Energy

**FIGURE 4:**
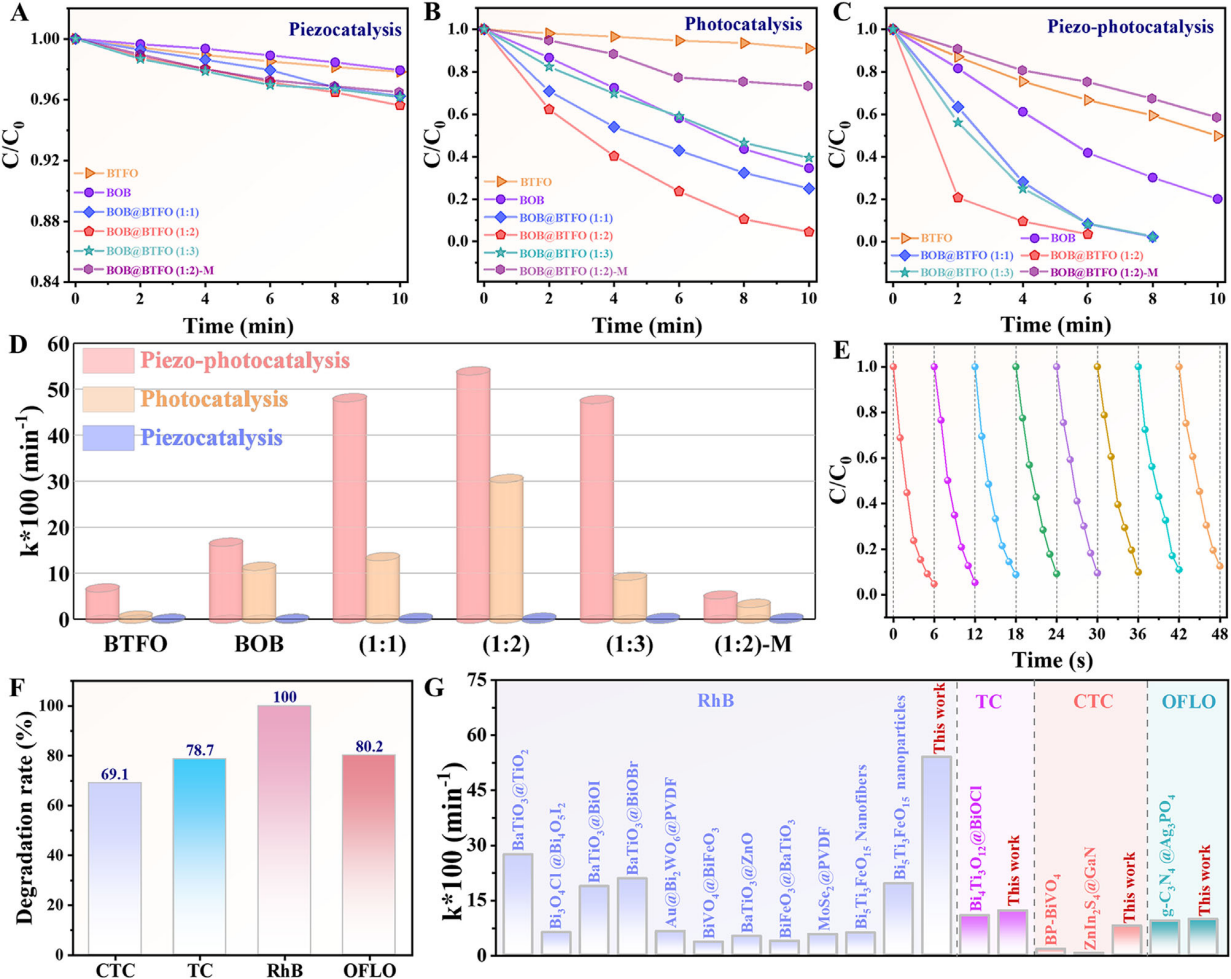
(A) Piezocatalytic, (B) photocatalytic, and (C) piezo‐photocatalytic degradation curves of RhB (concentration of 25 mg L^−1^) for BOB, BTFO, and BOB@BTFO, along with (D) the corresponding first‐order degradation rate constants. (E) Piezo‐photocatalytic cycling tests of BOB@BTFO (1:2). (F) Piezo‐photocatalytic performance of BOB@BTFO on various organic pollutants. (G) Comparison of piezo‐photocatalytic performance over BOB@BTFO with some piezo‐photocatalysts in previous reports.

To evaluate piezo‐photocatalytic activity, the degradation efficiency of RhB under ultrasonic vibration alone remained below 10% within 10 min (Figure 4A and Figure S8). Figure 4B and Figure S9 display the photocatalytic degradation profile of RhB for the samples, where BOB@BTFO (1:2) demonstrated near‐complete RhB removal (≈100%) within 10 min. Figure 4C and Figure S10 illustrate the piezo‐photocatalytic degradation profile of RhB for the samples. Although ultrasonic vibration alone showed limited contaminant degradation, the introduction of ultrasonic‐induced piezoelectric materials substantially enhanced the photocatalytic activity of BOB, BTFO, and BOB@BTFO. Notably, the BOB@BTFO (1:2) composite achieved 100% RhB removal within 6 min through piezo‐photocatalysis, exhibiting a reaction rate constant of 0.5399 min^−1^, which is 3.23 and 7.95 folds larger than that of BOB (0.1672 min^−1^) and BTFO (0.0679 min^−1^), respectively. Moreover, it surpasses the performance of individual photocatalysis (0.3057 min^−1^) and piezocatalysis (0.0042 min^−1^) by factors of 1.77 and 128.55, respectively (Figure 4D and Figure S11). The calculated synergy factors (SF) values for BOB, BTFO, BOB@BTFO (1:1), BOB@BTFO (1:2), and BOB@BTFO (1:3) are 6.06, 1.43, 3.44, 1.74, and 4.92, respectively (Figure S12). All SF values significantly exceed unity (SF > 1), unambiguously confirming the synergistic interaction between piezoelectric and photocatalytic mechanisms [40, 41]. Temperature‐dependent RhB degradation experiments conducted with BOB@BTFO (1:2) across 280–300 K revealed activation energies (*E*_a_) of 42.98 kJ mol^−1^ (photocatalysis), 52.13 kJ mol^−1^ (piezocatalysis), and 31.79 kJ mol^−1^ (piezo‐photocatalysis) through Arrhenius analysis (Figure S13A–D). The significantly reduced *E*_a_ in piezo‐photocatalysis provides direct evidence that piezoelectric polarization effectively lowers the reaction energy barrier through synergistic effects. Notably, the optimal molar ratio between BOB and BTFO components in the composite was identified as 1:2 through systematic optimization. In addition, the piezo‐photocatalytic performance of mechanically mixed BOB and BTFO (BOB@BTFO (1:2)‐M) was still worse compared to pure BOB and pure BTFO, which suggests the significance of heterojunction formation in enhancing the catalytic performance. The BOB@BTFO composite demonstrated exceptional total organic carbon removal efficiency, achieving 86.1% within a 6 min reaction (Figure S14). Furthermore, gas chromatography‐mass spectrometry analysis successfully identified intermediate products generated during RhB oxidation (Figure S15 and Table S2). Finally, the proposed degradation pathways of RhB were systematically illustrated in Figure S16.

The degradation performance of BOB@BTFO (1:2) remained stable through eight consecutive cycles of piezo‐photocatalytic experiments (Figure 4E). Post‐recycling characterization revealed no significant differences in crystalline structure or morphology between the initial and cycled samples (Figure S17A,B). XPS analysis further confirmed the structural integrity, with negligible shifts in the binding energies of Fe 2p, Ti 2p, and Br 3d between fresh and used catalysts (Figure S18). Additionally, the PFM measurements (Figure S19) revealed that the *d*_33_ of the BOB@BTFO (1:2) after long‐term reaction increased slightly compared to the pristine sample (from 60.65 pm V^−1^ increased to 68.95 pm V^−1^). These results collectively highlight the exceptional structural and mechanical stability of BOB@BTFO under piezo‐photocatalytic conditions. The BOB@BTFO exhibited broad‐spectrum pollutant degradation capabilities, achieving removal efficiencies of 69.1% for CTC, 78.7% for TC, and 80.2% for OFLO within 14 min under simultaneous ultrasound and light irradiation (Figure 4F and Figure S20). Comparative analysis with reported piezoelectric heterojunctions (e.g., BaTiO_3_@TiO_2_, BiFeO_3_@BaTiO_3_, MoSe_2_@PVDF) demonstrated the superior performance of BOB@BTFO, particularly in key metrics such as degradation efficiency, reaction rate, and pollutant concentration (Figure 4G and Table S3).

### 3.5 Adsorption Characteristics, Band Structure, Free Radical Capture, and Interface Charge Transfer Mechanism of 2D/2D Heterojunctions

**FIGURE 5:**
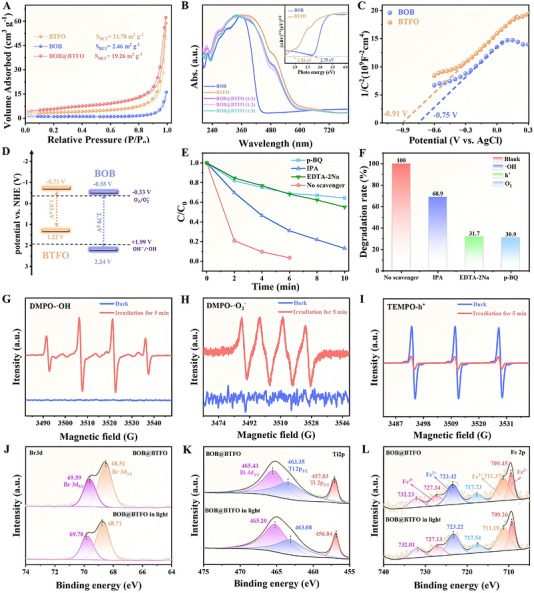
(A) N_2_ adsorption–desorption isotherms and (B) UV–vis diffuse reflectance spectra of BOB, BTFO, and BOB@BTFO, the inset shows Tauc plots. (C) Mott–Schottky curves and (D) band structure diagrams for BOB and BTFO. The piezo‐photocatalytic degradation performance of RhB for BOB@BTFO under (E) different radical scavengers and (F) the corresponding degradation efficiencies. ESR detection of (G) DMPO‐•OH, (H) DMPO‐•O_2_^−^, and (I) TEMPO‐h^+^ for the BOB@BTFO heterojunction. High‐resolution XPS spectra of BOB@BTFO: (J) Br 3d, (K) Ti 2p, and (L) Fe 2p with and without light irradiation.

The Brunauer–Emmett–Teller （BET） analysis of N_2_ adsorption–desorption isotherms and corresponding pore size distribution profiles (Figure 5A and Figure S21) revealed distinct specific surface areas: 19.26 m^2^ g^−1^ for BOB@BTFO, 2.46 m^2^ g^−1^ for BOB, and 11.78 m^2^ g^−1^ for BTFO. SEM/TEM characterization (Figure 1D–G,J,K) demonstrated that the uniform dispersion of BOB nanosheets on BTFO nanosheets creates three synergistic effects: (1) edge‐enriched surfaces, (2) inter‐nanosheet void spaces, and (3) anti‐agglomeration behavior during synthesis, collectively optimizing active site accessibility and enhancing specific surface area [42]. UV–vis diffuse reflectance spectra (Figure 5B) indicated a pronounced red‐shift in the absorption edge of BOB@BTFO compared to pristine BOB, accompanied by a minor blue‐shift relative to BTFO. Kubelka–Munk transformation yielded bandgap values of 2.79 and 1.93 eV for BOB and BTFO [43]. The flat band potentials (*E*_fb_) values based on Mott–Schottky plots BOB and BTFO calculated were −0.75 and −0.91 V (with respect to Ag/AgCl), and −0.55 and −0.71 V with respect to the normal hydrogen electrode (Figure 5C). Using the Nernst equation, the valence band potentials (*E*_VB_) for BOB and BTFO were calculated to be approximately 2.24 and 1.22 eV (vs. NHE), respectively, with the relationship defined as *E*_VB_ = *E*_CB _+ *E*_g_ [44]. The band structure of the BOB and BTFO aligned well with the thermodynamic oxidation–reduction potentials (Figure 5D). The *E*_VB_ of BTFO and BOB were also characterized by XPS valence spectra (Figure S22), and the BTFO and BOB were calculated to be about 1.27 and 2.25 eV, respectively, which are very close to those measured by the Mott–Schottky measurements.

The reaction mechanism of BOB@BTFO was investigated by radical trapping experiments during piezo‐photocatalytic degradation. As illustrated in Figure 5E,F, the RhB degradation efficiency dropped from 100% (no scavenger) to 68.9% with isopropyl alcohol (IPA, •OH scavenger), 31.7% with ethylenediaminetetraacetic acid disodium salt (EDTA‐2Na, h^+^ scavenger), and 30.9% with p‐benzoquinone (p‐BQ, •O_2_^−^ scavenger) within 6 min, demonstrating the dominant roles of h^+^ and •O_2_^−^, with •OH playing a secondary role. The potential generation of H_2_O_2_ during the piezo‐photocatalytic reaction process was quantitatively evaluated using the iodometric method [45, 46]. However, no distinct absorption signals corresponding to H_2_O_2_ were observed (Figure S23), indicating that H_2_O_2_ serves neither as a primary intermediate nor as a dominant reactive species in this system.

Electron spin resonance (ESR) spectroscopy using 5,5‐dimethyl‐1‐pyrroline *N*‐oxide (DMPO) and 2,2,6,6‐tetramethylpiperidine oxide (TEMPO) spin traps provided direct evidence of radical dynamics. No signals for DMPO‐•O_2_^−^ or DMPO‐•OH were observed under darkness (Figure 5G,H). After 5 min of illumination, a significant signal with a peak ratio of 1:2:2:1 confirmed the presence of •OH (Figure 5G). The DMPO‐•O_2_^−^ signal exhibited four broad peaks, indicating that oxygen was reduced by photoexcited electrons to form •O_2_^−^ (Figure 5H). Moreover, compared to dark conditions, the peak intensity of TEMPO significantly decreased after 5 min of light exposure, indicating h^+^ could be consumed in this reaction (Figure 5I). The main active radicals participating in the piezo‐photocatalytic degradation process were h^+^, •O_2_^−^, and •OH. The VB of BOB (2.24 eV vs. NHE) and the CB of BTFO (−0.71 eV vs. NHE) exhibited higher redox potentials compared to OH^−^/•OH (1.99 eV vs. NHE) and O_2_/•O_2_^−^ (−0.33 eV vs. NHE), confirming the formation of a Z‐scheme heterojunction between BOB and BTFO [47].

Furthermore, in situ irradiated XPS characterization was undertaken to validate the Z‐scheme charge transfer pathway between BOB and BTFO. In comparison to dark conditions, the binding energies of Br 3d in the BOB@BTFO under light irradiation shifted toward higher energy levels (Figure 5J), while the binding energies of Fe 2p and Ti 2p in the BOB@BTFO obviously moved to lower energy levels (Figure 5K,L), suggesting that photogenerated electrons can transfer from BOB to BTFO under light irradiation [42]. In situ irradiated XPS further revealed that the BOB@BTFO heterojunction exhibited markedly larger binding energy shifts for Br (BOB) and Fe/Ti (BTFO) compared to single BOB and BTFO (ΔBE < 0.04 eV, Figure S24), underscoring enhanced interfacial charge redistribution at the heterojunction. While analogous shifts were observed for O 1s and Bi 4f (Figure S25), the shared presence of these elements in both BOB and BTFO precludes definitive assignment of charge origins.

### 3.6 Piezoelectric Polarization‐Induced Photogenerated Carriers Transfer Mechanism

**FIGURE 6:**
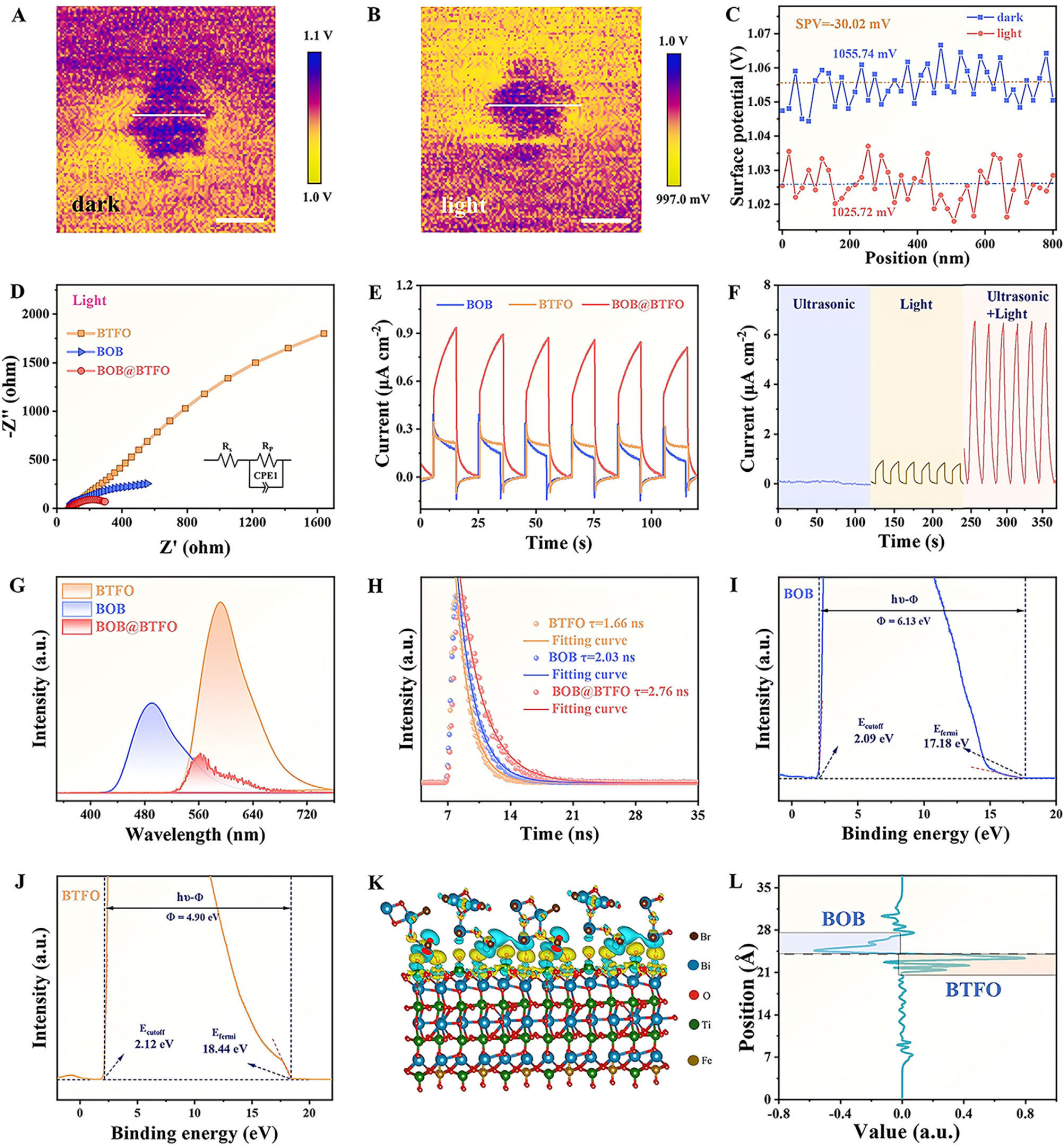
KPFM potential images of BOB@BTFO nanosheet (A) under the darkness and (B) light illumination, (C) the corresponding surface potential profiles along lines in (A) and (B), the scale size was 500 nm. (D) The EIS Nyquist plots in light and (E) photocurrent response of BOB, BTFO, and BOB@BTFO under light illumination. (F) Piezoelectric, photocurrent, and piezo‐photocurrent responses of BOB@BTFO. (G) Steady‐state PL spectra, and (H) time‐resolved transient PL spectra. UPS spectra of (I) BOB and (J) BTFO. (K) The charge density difference distribution of BOB@BTFO, and (L) the planar averaged electron density difference (Δρ).

To probe photogenerated charge carrier dynamics, Kelvin probe force microscopy (KPFM) was employed to quantify the surface photovoltage (SPV) of BOB@BTFO nanosheets. Figure 6A–C displays KPFM topographic images and corresponding surface potential distributions under dark and illuminated conditions. A decrease in surface potential was observed under illumination, with the average value decreasing from 1055.74 mV (dark) to 1025.72 mV (light), corresponding to an SPV of −30.02 mV. This negative SPV value confirms the preferential migration of photogenerated electrons to the BOB@BTFO surface, facilitated by the built‐in interfacial electric field [48].

Electrochemical impedance spectroscopy (EIS) measurements demonstrated minimal charge transfer resistance, as evidenced by its smallest Nyquist arc radius relative to pristine BOB and BTFO under both dark and illuminated conditions (Figure 6D, Figure S26, and Table S4). Additionally, the photocurrent density of the BOB@BTFO was significantly higher than that of pure BOB and pure BTFO (Figure 6E), which was in agreement with the results of EIS. Moreover, the current response of BOB@BTFO under simultaneous illumination and ultrasonic vibration was significantly higher than that observed with either light or ultrasound alone (Figure 6F), demonstrating a synergistic effect between the interfacial electric field and ultrasonic‐induced piezoelectric polarization. Steady‐state photoluminescence (PL) spectra in Figure 6G revealed a weaker intensity of the PL for the BOB@BTFO heterojunction compared to pure BOB and pure BTFO, suggesting effective suppression of photo‐generated electron–hole recombination. Time‐resolved PL decay kinetics fitted with a biexponential model (Figure 6H) further quantified charge dynamics: the heterojunction exhibited an extended average carrier lifetime (2.76 ns) versus BOB (2.03 ns) and BTFO (1.66 ns), confirming enhanced charge transfer efficiency [49].

Moreover, ultraviolet photoelectron spectroscopy (UPS) spectra were employed to analyze the work functions (*Φ*) of BOB and BTFO, as shown in Figure 6I,J. The *Φ* for BOB and BTFO were estimated to be 6.13 and 4.90 eV, respectively, corresponding to their respective Fermi levels (*E*_f_) [50]. The substantial work function difference drives interfacial charge transfer, with electrons spontaneously migrating from low‐*Φ* BTFO to high‐*Φ* BOB until Fermi level equilibration. This redistribution establishes a positively charged BTFO interface with upward band bending and a negatively charged BOB interface exhibiting downward band bending, creating electron accumulation zones. Consequently, a built‐in electric field (IEF) oriented from BTFO to BOB forms at the heterointerface. Under irradiation, the photogenerated electrons accumulated in the CB of BOB would flow to the VB of BTFO under the driving force of the internal electric field at the interface of BOB and BTFO, forming a Z‐scheme charge transfer mechanism (Figure S27).

Additionally, the calculated difference in average electron density (Δ*ρ*) of BOB@BTFO heterojunction (Figure 6K,L) indicated charge migration at the interface between BOB and BTFO, leading to electron redistribution. Figure 6K illustrates the lateral charge distribution difference of BOB@BTFO, where light blue and yellow represent the depletion region and accumulation region of the electron, respectively. The surface of BTFO was covered with yellow color, while the side of BOB was characterized by light blue color, which visualized that the BTFO acquired electrons from the BOB side and induced the generation of an internal electric field in the heterostructure. In addition, the plane‐average electron density difference along the *Z* direction (Figure 6L) also indicated that BTFO absorbed electrons from the BOB in the absence of light.

### 3.7 Z‐Scheme Charges Transfer Mechanism Regulated by Dual‐Piezoelectric Field

**FIGURE 7:**
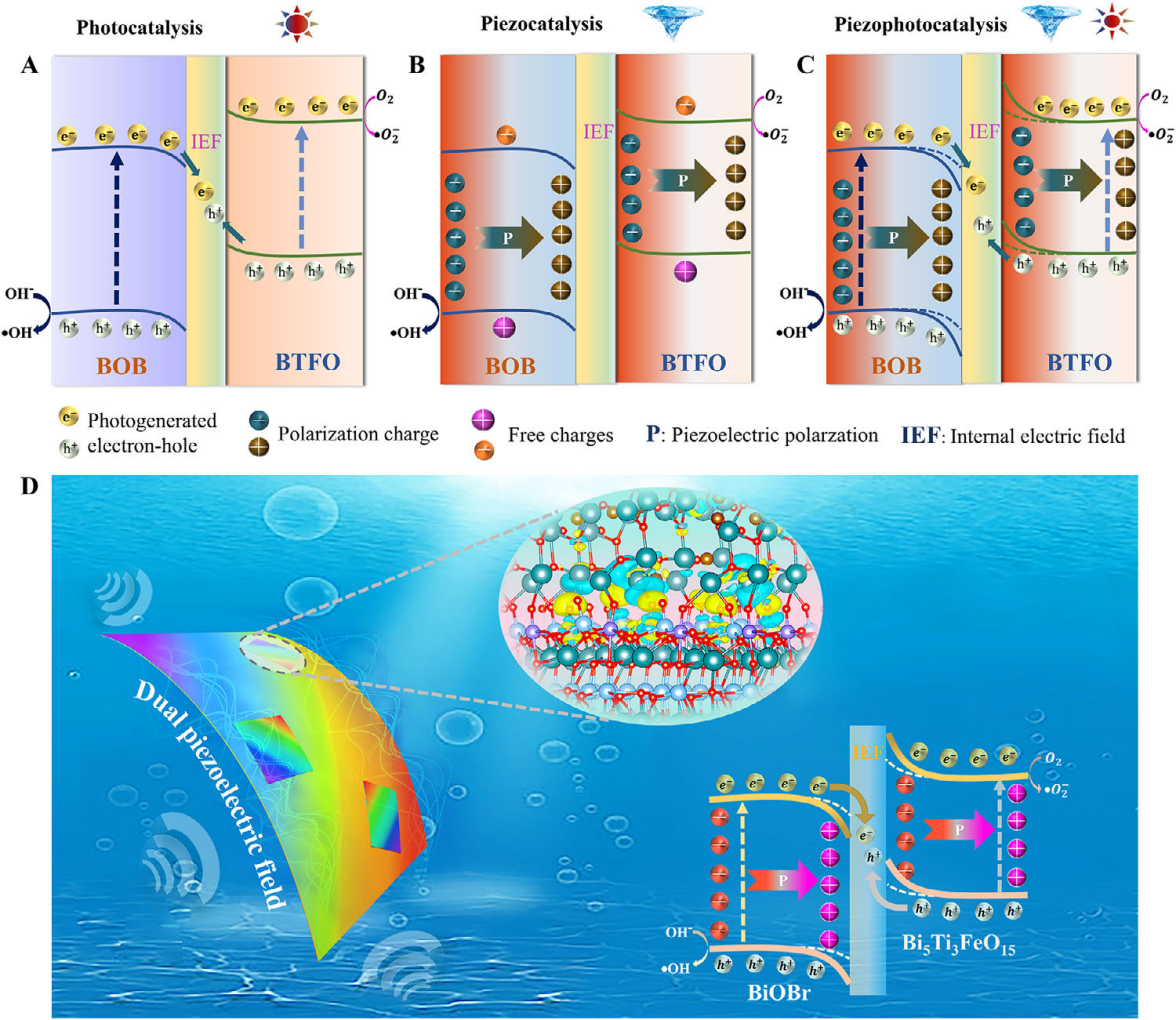
Schematic representation of the BOB@BTFO heterojunction under (A) illumination, (B) ultrasound, and (C) combined illumination‐ultrasound conditions. (D) Schematic diagram of BOB@BTFO nanosheets promoting piezo‐photocatalytic degradation.

Based on the aforementioned results and discussions, as illustrated in Figure 7A, both BOB and BTFO are photoexcited to generate electron–hole pairs under light illumination without ultrasound. The remnant polarization in BTFO induces a depolarization field, which drives the migration of electrons from the CB of BOB to the VB of BTFO. Consequently, high‐energy electrons and holes remain localized in the CB of BTFO and the VB of BOB, respectively. This mechanism enables BOB@BTFO to achieve a degradation rate significantly surpassing that of non‐ferroelectric BOB. Under ultrasound without light, the piezocatalytic rate of BOB@BTFO, BTFO, and BOB correlates with strain‐driven piezoelectric polarization (Figure 3H–M). During the piezo‐catalytic process, piezoelectric polarization electric fields were generated on both sides of the BOB and BTFO under ultrasonic vibration, resulting in band bending, which drove the migration of free charge in opposite directions and reached the catalyst surface to participate in the reduction and oxidation reactions (Figure 7B). However, the piezoelectric effect produced a relatively low concentration of free charges, limiting the overall activity of the catalytic degradation process. Under the combined excitation of ultrasonic vibration and light, the separation of photoinduced charge carriers was significantly enhanced by the ferro‐/piezo‐electric polarization field and band bending, as illustrated in Figure 7C. In contrast to traditional Z‐scheme heterojunction electron transfer systems, the interfacial electric field induced by the ferro‐/piezo‐electric effect will further modulate the interfacial energy band bending. Specifically, as the CB of BOB bends downward and the VB of BTFO bends upward, the bands tilt further under the influence of the piezoelectric potential, thereby enhancing the separation efficiency of bulk and interface charge carriers. Simultaneously, photoinduced electrons and holes are transported to the surfaces of BOBs and BTFOs, respectively, under the interfacial electric field induced by piezoelectric polarization. This promoted a greater involvement of photogenerated carriers in the activation of O_2_, resulting in the generation of •O_2_^−^ and •OH. Moreover, the piezoelectric potential exhibited periodic fluctuations as a function of ultrasound, which mitigated the shielding effect of the depolarizing field in BOB and BTFO. Furthermore, the extensively exposed two‐dimensional layered structures of BOB and BTFO serve as reactive sites for the degradation of organic pollutants, thereby reducing activation barriers. This synergistic interplay between piezoelectric and photocatalytic effects facilitated the spatial separation of charge carriers, allowing them to migrate to the catalyst surface of BOB@BTFO, which boasted abundant exposed active sites (Figure 7D). Consequently, this significantly enhanced the piezo‐photocatalytic activity for the degradation of organic pollutants.

## 4 Conclusion

In conclusion, the inter‐plane 2D/2D BOB@BTFO heterojunction with dual piezoelectric response was successfully fabricated for piezo‐photocatalytic degradation through in situ growth of BOB nanosheets—sharing analogous [Bi_2_O_2_]‐based layered structures—on BTFO nanosheets. The PFM, DFT calculation, and COMSOL simulations collectively revealed that the atomic‐level interfacial chemical bonds (Bi‐OTi and Bi‐O‐Fe bonds) and strain‐induced electronic coupling synergistically enhance piezoelectric properties, achieving a piezoelectric coefficient (*d*_33_) of 60.65 pm V^−1^. The piezo‐photocatalytic activity of all materials was assessed through degrading RhB, and optimized BOB@BTFO exhibited an excellent piezo‐photocatalytic degradation rate (0.5399 min^−1^), which was significantly greater than that of the pure BOB (0.1672 min^−1^) and pure BTFO (0.0679 min^−1^), as well as higher than that of photocatalytic (0.3057 min^−1^) and piezocatalytic (0.0042 min^−1^) alone. Such excellent piezo‐photocatalytic activity is ascribed to the synergistic effect between the dual‐piezoelectric field and the atomic‐level heterojunction engineering, which not only regulates the interfacial band bending but also provides an interfacial electric field to drive photogenerated charge migration and redox kinetics. Moreover, the detection of active species radicals (•O_2_^−^, h^+^, and •OH) during the piezo‐photocatalytic degradation of RhB confirmed the Z‐scheme charge transfer mechanism. The proposed 2D/2D [Bi_2_O_2_]‐based ferro‐/piezo‐electric heterojunction demonstrates excellent dual piezoelectric photocatalytic performance, providing an innovative strategy for optimizing catalytic technology and broadening its application prospects in environmental remediation and energy conversion.

## Author Contributions

**Daotong You**: methodology, supporting, supervision, discussion, formal analysis, writing – original manuscript, writing – review and editing. **Xingwang Long**: conceptualization, data curation, writing – original manuscript. **Zhiyong Yang**: software, investigation, formal analysis. **Lei Liu**: software, investigation, formal analysis. **Jianbang Chen**: visualization, investigation. **Tuan Guo**: conceptualization, funding acquisition, supporting.

## Conflicts of Interest

The authors declare no conflicts of interest.

## Supporting information




**Supporting File**: exp270169‐sup‐0001‐SuppMat.docx.

## Data Availability

Data are available from the corresponding author upon reasonable request.
