# Supplementary material for: Dual Ferro‐/Piezo‐Electric Coupling in Two‐Dimensional [Bi2O2]‐Based Layered Structures for Synergistic Harvesting of Mechanical and Solar Energy
Source: Exploration (Beijing). 2026 May 8;6(3):20250105. doi: 10.1002/EXP.20250105 (PMC13317668; doi:10.1002/EXP.20250105)
Supplement: Supplementary file 1 — Supporting File: exp270169‐sup‐0001‐SuppMat.docx. [file EXP2-6-20250105-s001.docx]

**Supporting Information**

**Dual ferro-/piezo-electric coupling in two-dimensional [Bi_2_O_2_]-based layered structures for synergistic harvesting of mechanical and solar energy**

*Daotong You^#, *,1,2^, Xingwang Long^#,1^, Zhiyong Yang^1^,* *Lei Liu^1^, Jianbang Chen^1^ and Tuan Guo^1^*

1. Institute of Photonics Technology, College of Physics and Optoelectronic Engineering,

Jinan University, Guangzhou 510632, Peoples Republic of China

1. School of Optoelectronic Engineering, Guangxi Key Laboratory of Optoelectronic Information Processing, Guilin University of Electronic Technology, Guilin 541004, Peoples Republic of China

* Corresponding E-mail addresses: [youdaotong@jnu.edu.cn](mailto:youdaotong@jnu.edu.cn)

^#^ These authors contributed equally: Daotong You and Xingwang Long

**1.Materials**

Bismuth nitrate pentahydrate (Bi(NO_3_)_3_·5H_2_O, AR, 99%); Potassium bromide (KBr, metals basis, 99.95%); Ethanol (99.7%); Ethylenediaminetetraacetic acid disodium salt (EDTA-2Na, AR,99%); P-benzoquinone (p-BQ, 99%); Isopropyl alcohol (IPA, AR); 5,5-dimethyl-1-pyrroline N-oxide (DMPO, AR); 2,2,6,6-tetramethylpiperidine oxide (TEMPO, AR); Rhodamine B (RhB, AR) and Sodium sulfate (Na_2_SO_4_, AR, 99.99%) were purchased from Shanghai McLean Biochemical Technology Co., LTD. Tetrabutyl titanate (Ti(C_4_H_9_O)_4_, ≥98%) and sodium hydroxide (NaOH, AR, ≥98%) were purchased from Aladdin Biochemical Technology Co., LTD. Iron nitrate (Fe(NO_3_)_3_·9H_2_O, 99.99%) was purchased from Shanghai Rin En Technology Development Co., LTD. Potassium ferricyanide (C_6_FeK_3_N_6_, 99.95%), purchased from Shanghai Yien Chemical Technology Co., LTD.

**2. Characterization**

The phase of the material was analyzed using X-ray diffraction (XRD, Bruker D8 Advance) with Cu Kα radiation (40 kV and 40 mA). Raman spectra at room temperature were recorded using a micro-Raman spectrometer (Thermo Scientific DXRTM) with a 785 nm laser as the excitation source, employing a 50× objective lens. The sample morphology was analyzed using a Scanning Electron Microscope (SEM, Hitachi SU8010) and a Transmission Electron Microscope (TEM, JMF-2100F). The microstructure and elemental distribution of the sample were characterized using High-Angle Annular Dark Field Scanning Transmission Electron Microscopy (HAADF-STEM, JMF-2100F) and Energy Dispersive X-ray Spectroscopy (EDX). The Brunauer-Emmett-Teller (BET, Quantachrome) method was employed to calculate the specific surface area and pore size analysis via N_2_ adsorption-desorption. Surface electronic states were assessed through X-ray photoelectron spectroscopy (XPS, Thermo Scientific K-Alpha), with all binding energies calibrated against C1s (284.6 eV). UV-visible diffuse reflectance spectra (DRS) were obtained using a UV-Visible spectrophotometer (UV3600, Agilent) with BaSO_4_ as the reflection standard. Atomic force microscopy (AFM) (Bruker Multimode 8, Germany) was utilized to study the morphology and thickness of the samples, with piezoelectric force microscopy (PFM) and Kelvin probe force microscopy (KPFM) modules employed to explore piezoelectric characteristics and surface potential, respectively. The height, amplitude, phase images, and PFM images were obtained through contact mode measurements. The polarization and electric field curves (P-E hysteresis loop) of samples were characterized with an electric hysteresis loop measuring instrument (ACIX TF2000E) at a frequency of 1 kHz. The valence band maxima (VBM) were determined by linearly extrapolating the leading edge of the XPS valence band spectra to the baseline, and based on the conversion formula:

$E_{VB,NHE}= \varphi+ E_{VB,XPS}-4.44$ (1)

where φ is the electron work function of the analyzer (4.20 eV).

The ultraviolet photoelectron spectrum (UPS, 250Xi, Thermo Fisher Scientific, USA) was measured to determine the work function of the sample. The Φ is estimated by secondary electron cut-off (E*_cutoff_*), Fermi edge spectrum (E*_fermi_*)and the Ultraviolet photoelectron spectroscopy (UPS) following the equation:

$\Phi=h\nu-(E_{cutoff}-E_{fermi})$ (2)

where hν is the excitation energy from photon source He-Ⅰ (21.22 eV).

Steady-state Photoluminescence (PL) was conducted using a Hitachi F-7000 spectrophotometer with an excitation wavelength of 370 nm, while time-resolved transient PL (TRPL) was measured using an FLS980 spectrophotometer. The electron paramagnetic resonance (EPR) spectral data were recorded by a spectrometer (Bruker A300). Electron spin resonance (ESR) analysis was performed with 5,5-dimethyl-1-pyrroline N-oxide (DMPO) and 2,2,6,6-tetramethylpiperidine oxide (TEMPO) (A300, Bruker, Germany) to detect signals of hydroxyl radicals (•OH), superoxide anion radicals (•O_2_^-^), and holes (h⁺), respectively.

To investigate the electrochemical properties of the photocatalyst, transient photocurrent response (I-T), electrochemical impedance spectroscopy (EIS), and Mott-Schottky (M-S) analysis were conducted in a 0.5 mol L^-1^ Na_2_SO_4_ solution using an electrochemical workstation (CHI660D) configured in a three-electrode system. This system comprised an Ag/AgCl reference electrode, a Pt line pair counter electrode, and a working electrode. Especially, the working electrode was fabricated by uniformly mixing 5 mg photocatalyst in 300 μL ethanol and 15 μL Nafion, then coating 60 μL slurry sample on 1 cm^2^ surface of indium tin oxide (ITO) conductive glass and drying at 60°C for 1 h. In addition, PLS-SXE300D xenon lamp was used as the light source for photocurrent response measurement.

**3. Catalytic performance test**

The degree of contaminant mineralization was assessed by determining the total organic carbon (TOC) content with a TOC analyzer (TOC-VCPN, Shimadzu, Japan). Gas Chromatography-Mass spectrometry (GC7890/MS5975, Agilent Technologies, USA) was used to identify the intermediate products generated during the oxidation of RhB.

To identify the active species involved in the photocatalytic process, reactive species trapping experiments were conducted. Specifically, p-benzoquinone (p-BQ) (0.2 mM), ethylenediaminetetraacetic acid disodium salt (EDTA-2Na) (0.2 mM), and isopropanol (IPA) (10 mM) were added to the aforementioned mixture as scavengers for superoxide radicals (•O_2_⁻), holes (h⁺), and hydroxyl radicals (•OH), respectively.

We performed quantitative H₂O₂ detection using the iodometric method, which involved mixing 1 mL solution after piezo-photocatalytic process by BOB@BTFO with 1 mL of 0.4 M potassium iodide and 1 mL of 0.1 M potassium hydrogen phthalate solutions, followed by monitoring the absorbance by UV-vis spectroscopy at ambient conditions at 350 nm.

The reaction rate constants (k) were calculated using pseudo-first-order kinetics, as expressed in Equation (3):

$-In \frac{C}{C_{0}} = kt$ (3)

where C_0_ is the initial absorbance of RhB and C is the absorbance at any time (t) during the measurements, while k is the apparent rate constant from the degradation of RhB.

The Arrhenius equation (Ea) and Eyring analysis to extract the activation energy from the aerobic RhB degradation and thermodynamic parameters, respectively, as demonstrated in Equations (4):

$Ink = \frac{-Ea}{\mathrm{RT}} + InA$ (4)

where Ea (kJ mol^−1^) is the activation energy from the RhB degradation, T (K) is the experiment temperature, R is a universal gas constant, lnA is the prefactor.

The synergy factors (SF) are calculated using Equation (5) based on the reaction rate constants derived from piezophotocatalytic (k_piezo-photo_) piezocatalytic (k_piezo_), and photocatalytic (k_photo_) processes:

$SF = \frac{k(\mathrm{piezo}-\mathrm{photo})}{k(photo)+k(piezo)}$ (5)


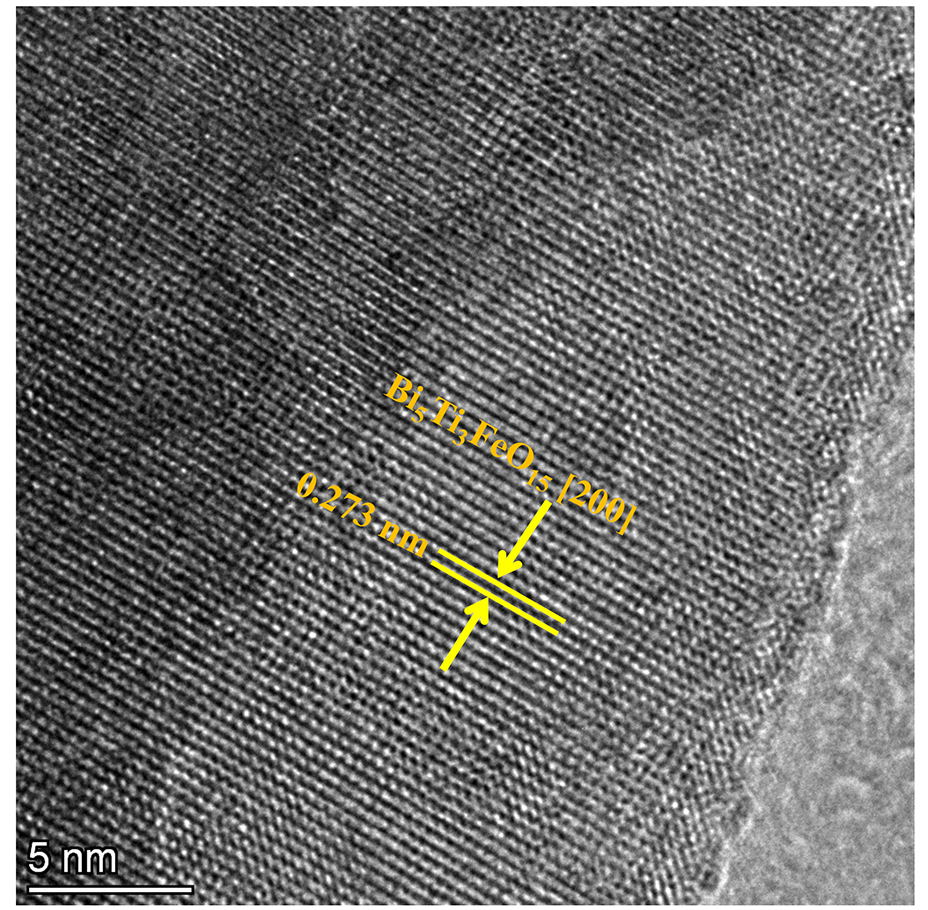


**Figure S1.** HRTEM images of BTFO nanosheet.


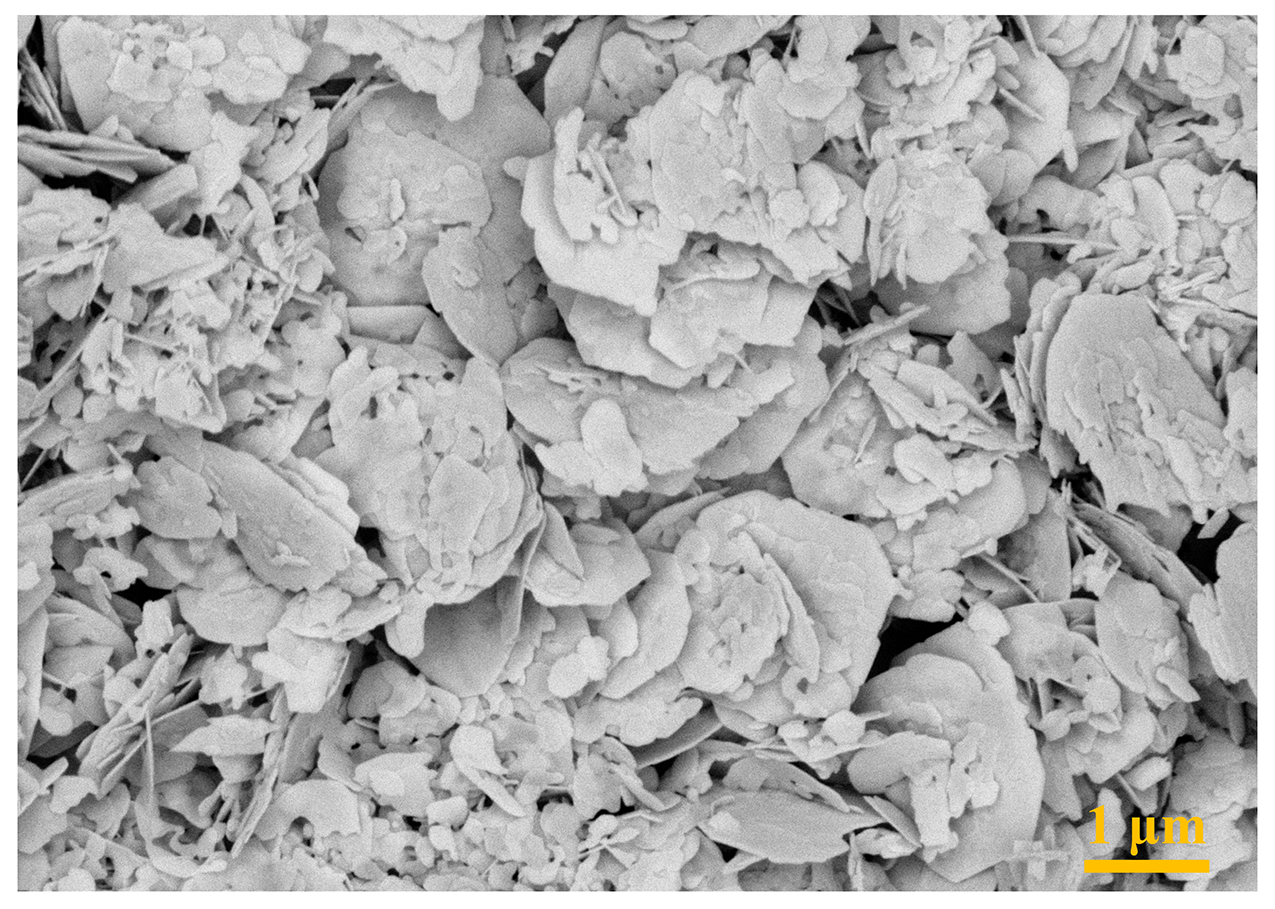


**Figure S2.** SEM images of BOB nanosheets.


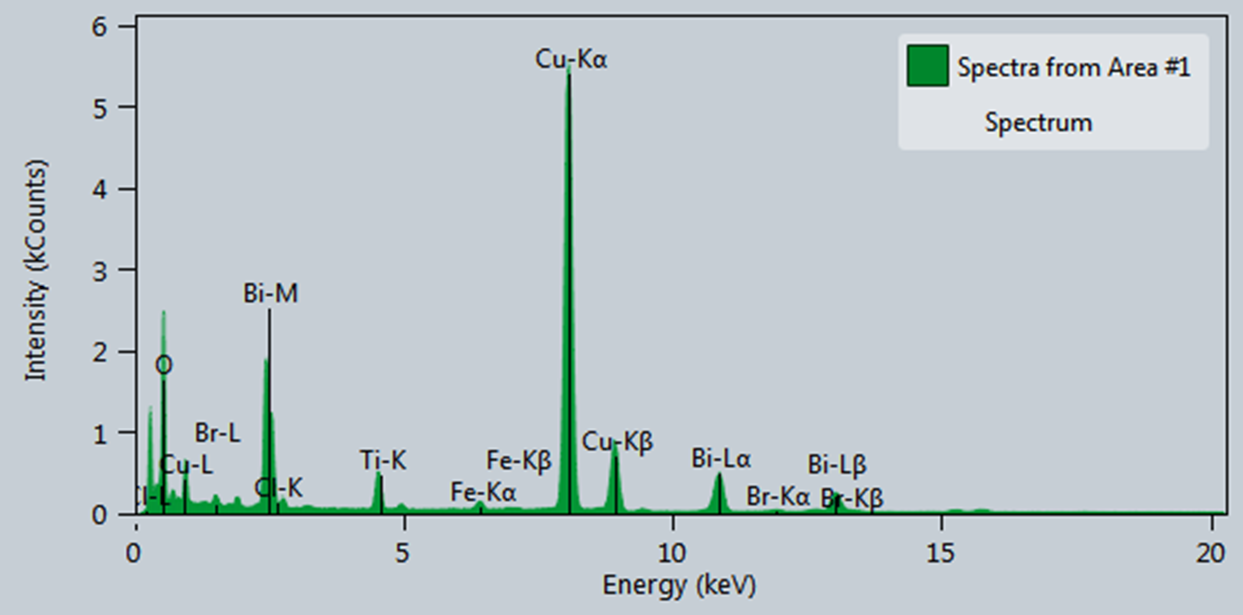


**Figure S3.** The elemental distribution within the BOB@BTFO nanosheets heterojunction.


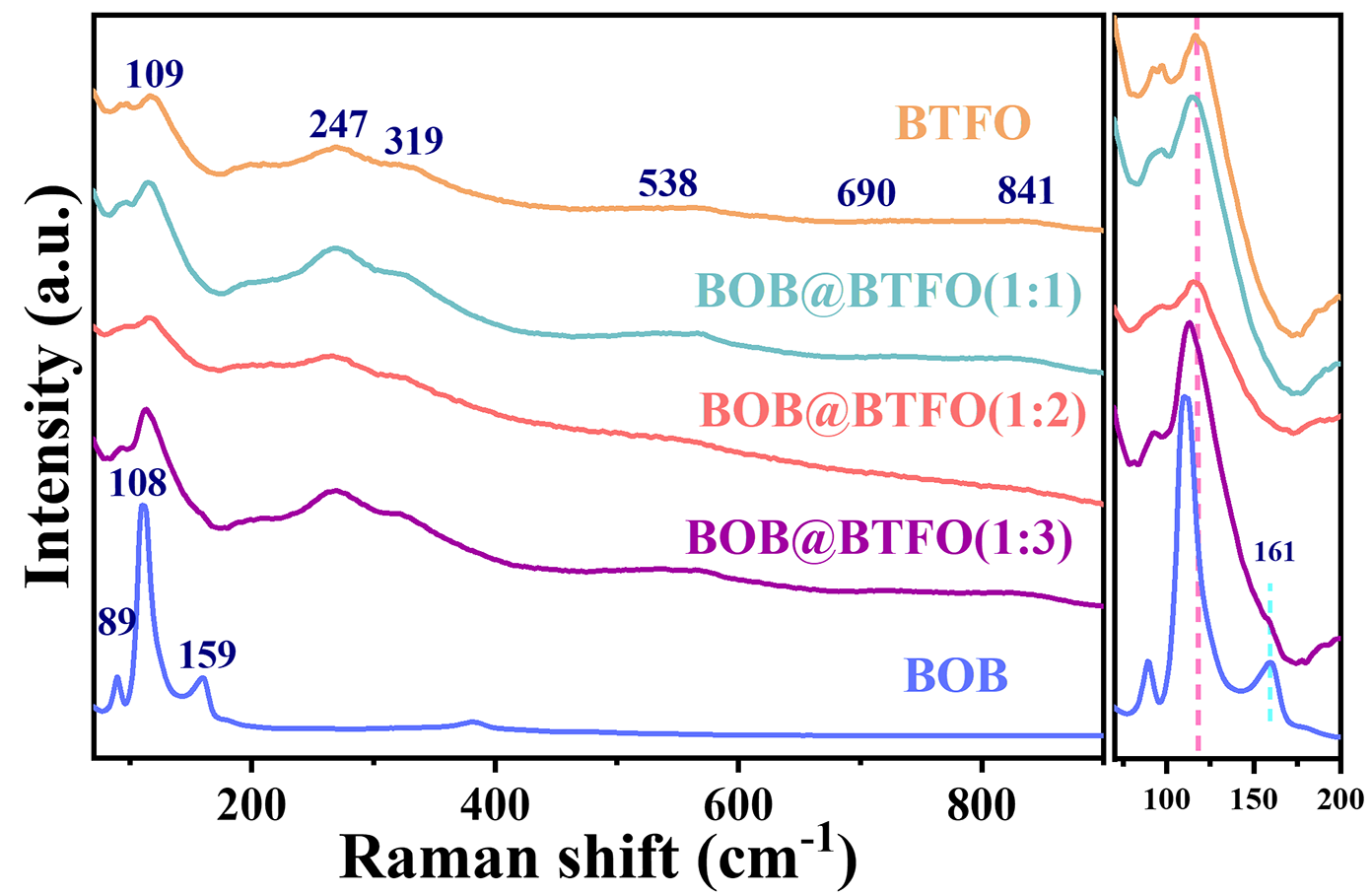


**Figure S4.** Raman spectra of BOB, BTFO and their various ratios BOB@BTFO.

As shown in **Figure S4**, the Raman spectrum of BTFO revealed phonon modes at 109, 247, 319, 538, 690, and 841 cm⁻¹. The modes around 247, 319, 538, and 841 cm⁻¹ were associated with internal modes of the A1g characteristic TiO6 octahedra. Specifically, the modes at 247 cm⁻¹ and 841 cm⁻¹ corresponded to O-Ti-O torsional vibrations and O-Ti stretching vibrations, respectively. The mode at 319 cm⁻¹ was related to the coupled torsional and stretching vibrations of the oxygen octahedra. Additionally, the mode at 109 cm⁻¹ arised from the vibrations of the A-site Bi ions within the perovskite lattice, while the peak at 690 cm⁻¹ was linked to the FeO6 octahedra. Raman peaks located at 89, 108, and 159 cm⁻¹ are attributed to the external A1g Bi–Br stretching mode, the internal A1g Bi–Br stretching mode, and the E1g internal Bi–Br stretching mode, respectively.


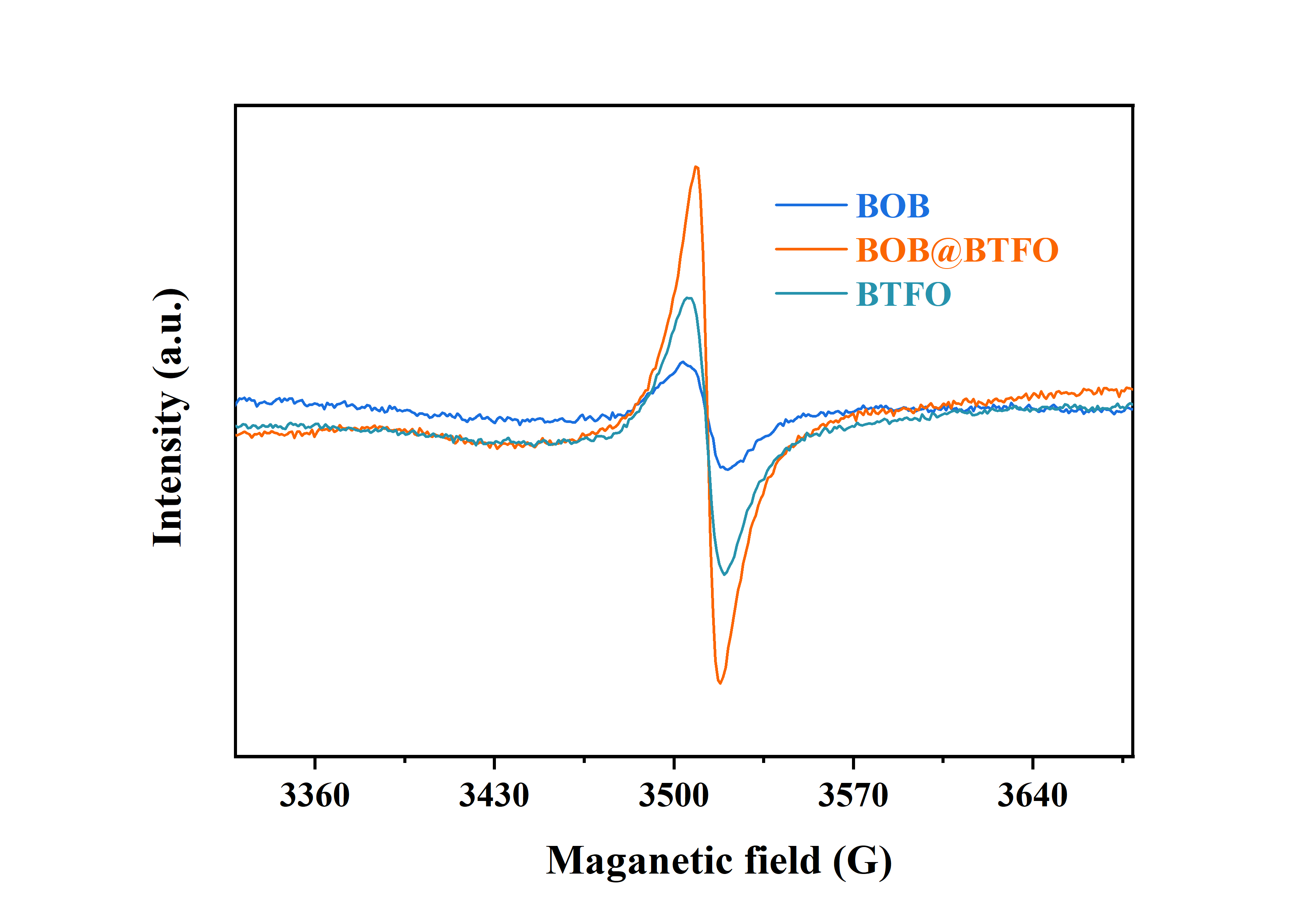


**Figure S5**. EPR spectra of pristine BOB, BTFO, and BOB@BTFO heterojunction.


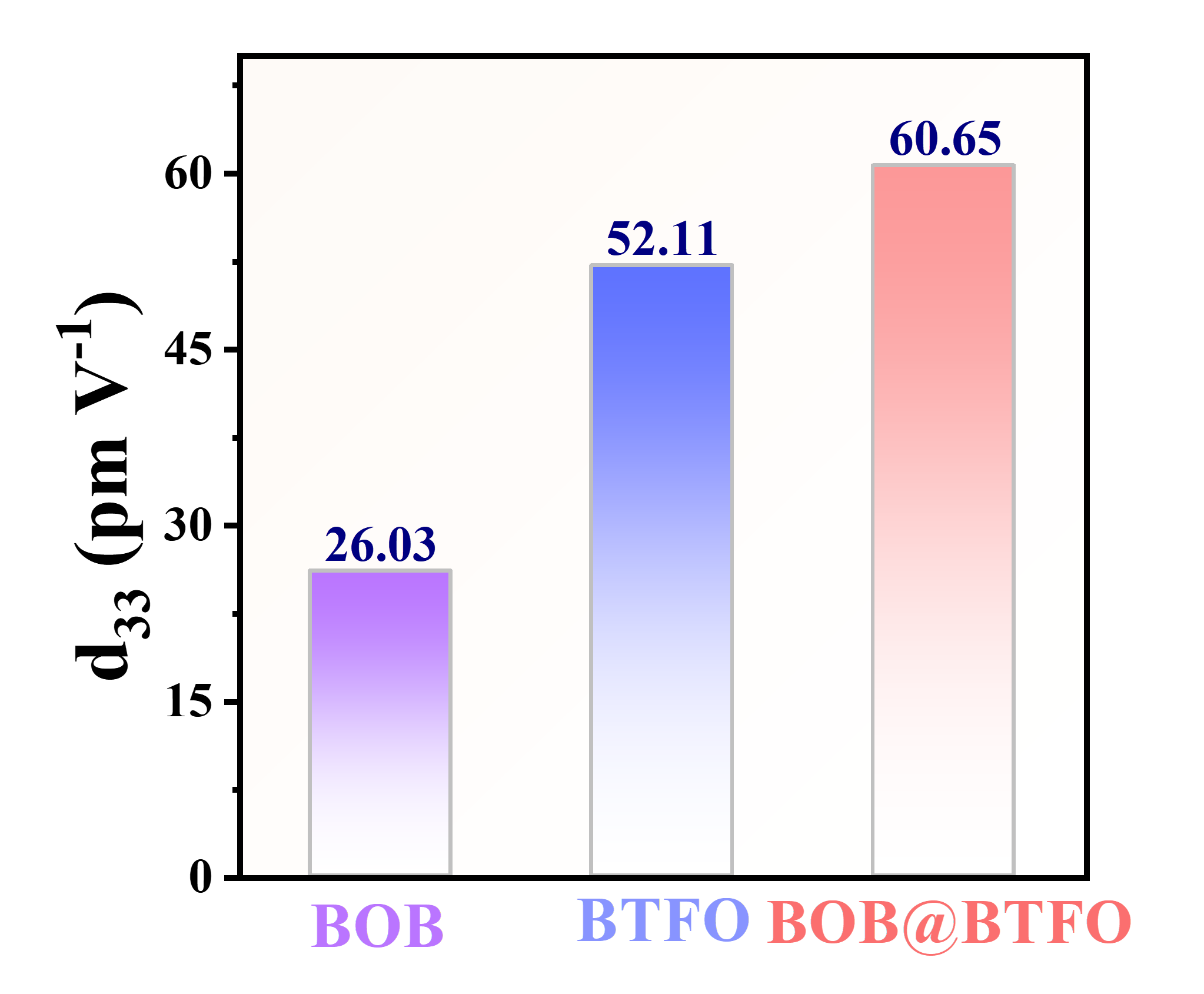


**Figure S6.** d_33_ values of BOB, BTFO, and BOB@BTFO.

**Table S1**. comparison data of the piezoelectric coefficient (d_33_) of BOB@BTFO with reported piezoelectric heterojunctions

| **Catalysts** | **d_33_ (pm/V)** | **Ref.** |
| --- | --- | --- |
| BaTiO_3_ nanoparticles@TiO_2_ micronflowers | 6.5 | [1] |
| Au@Bi_2_WO_6_ microspheres @PVDF films | 20 | [5] |
| needle-like BaTiO_3_@ZnO nanofibers | 44.5 | [7] |
| BiFeO_3_ nanoparticles@BaTiO_3_ nanofibers | 15.3 | [8] |
| MoSe_2_ nanoparticles@PVDF films | 6.5 | [9] |
| MoS_2_ nanosheets@g-C_3_N_4_ nanosheets | 16.3 | [16] |
| Sr-doped Bi_4_O_5_Br_2_@Bi_2_MoO_6_ nanofibers | 21.7 | [17] |
| BiOBr nanosheets @Bi_5_Ti_3_FeO_15_ nanosheets | 60.65 | This work |


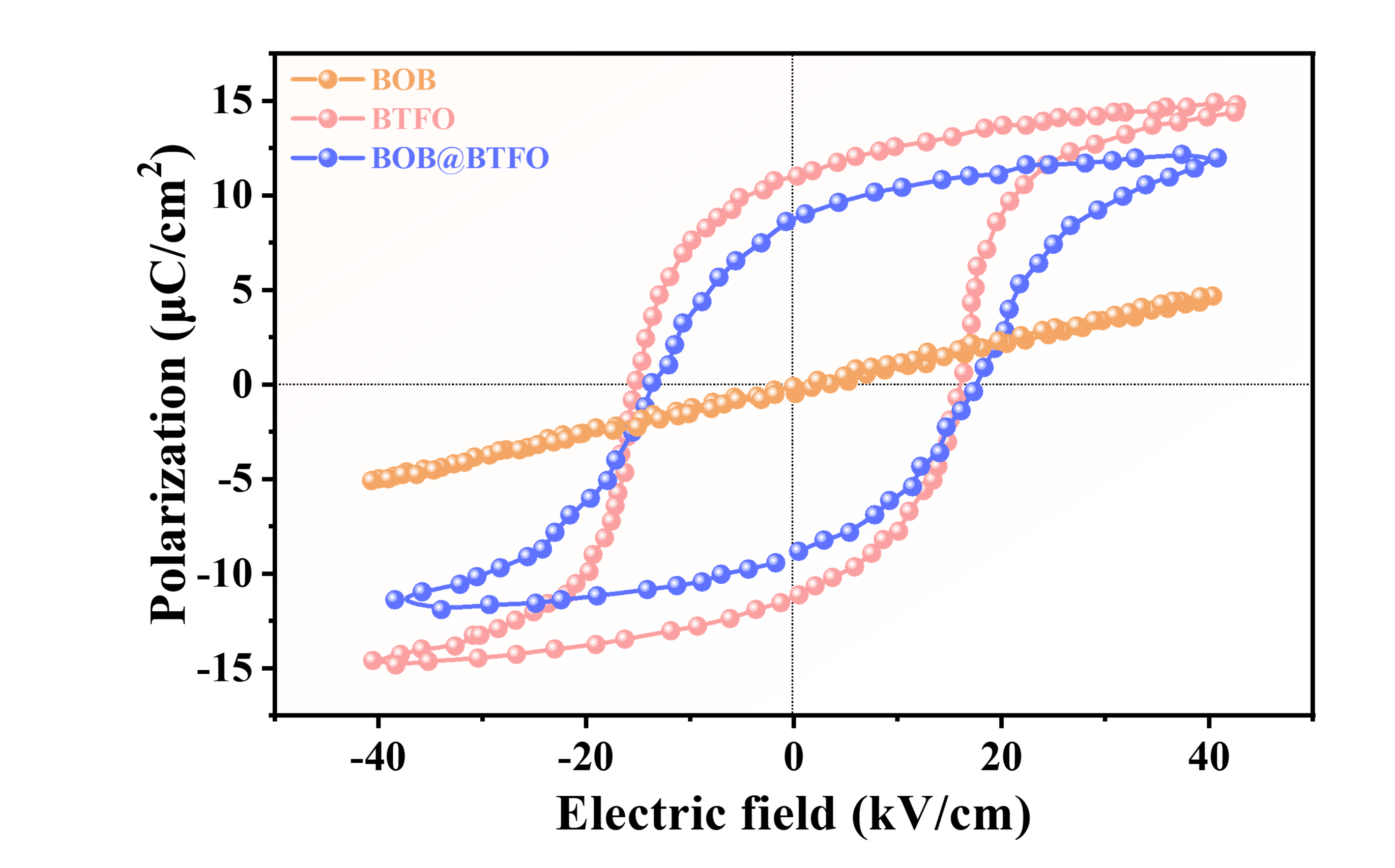


**Figure S7.** Ferroelectric hysteresis loop of BOB, BTFO, and BOB@BTFO.


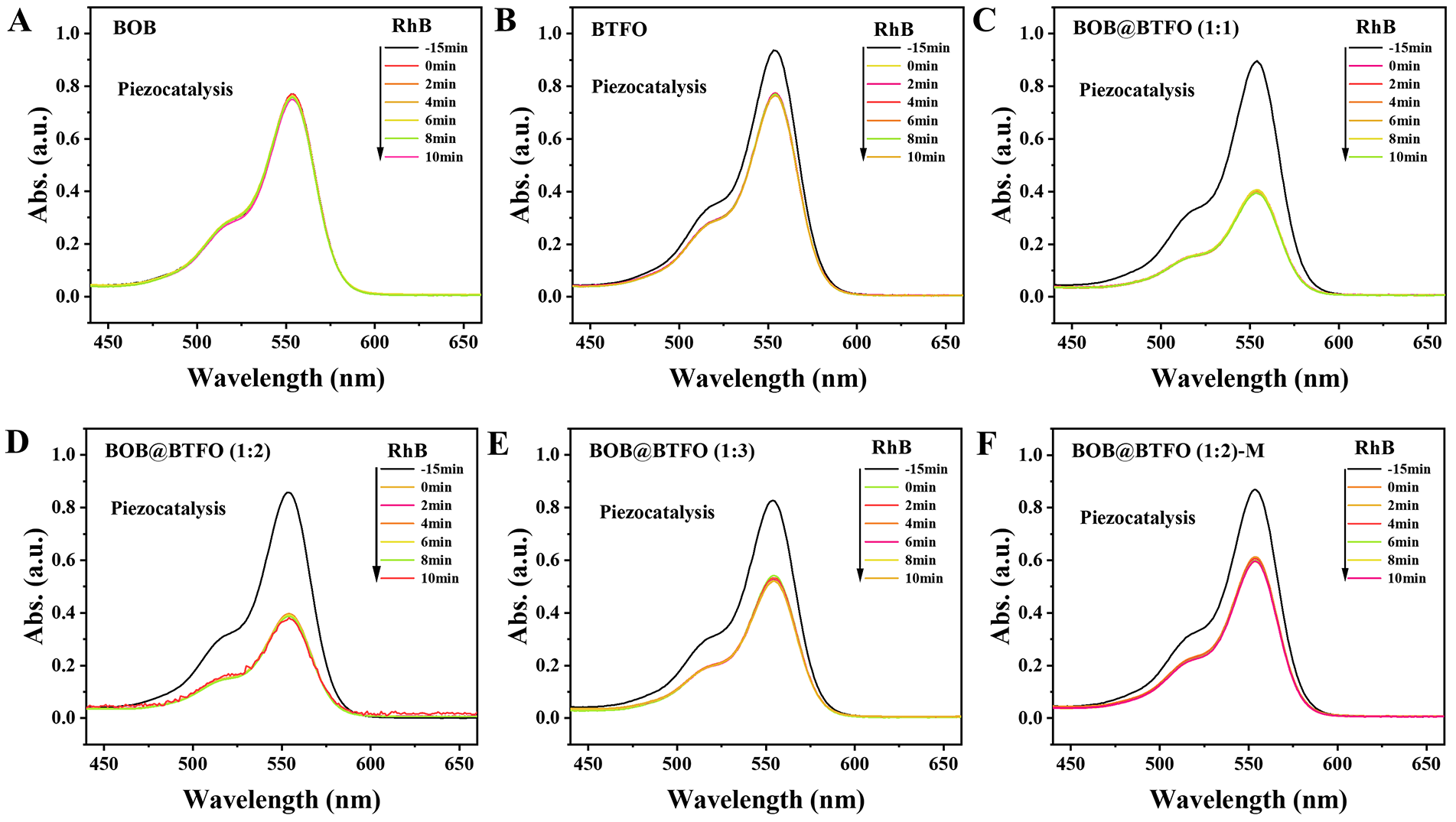


**Figure S8.** Absorption spectrum of (A) BOB, (B) BTFO, (C) BOB@BTFO (1:1), (D) BOB@BTFO (1:2), (E) BOB@BTFO (1:3) and (F) BOB@BTFO (1:2)-M samples during piezocatalytic degradation of RhB.


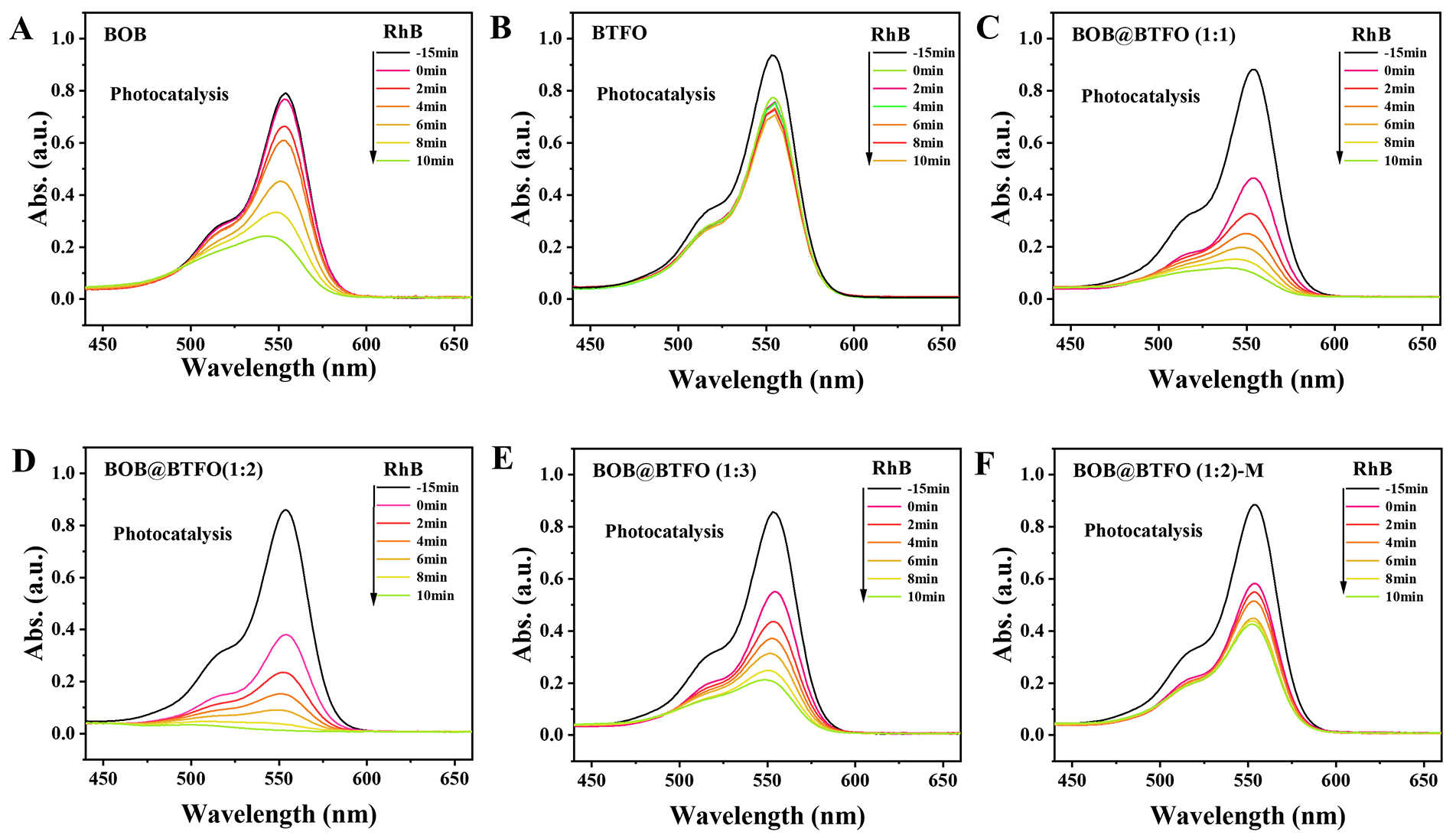


**Figure S9.** Absorption spectrum of (A) BOB, (B) BTFO, (C) BOB@BTFO (1:1), (D) BOB@BTFO (1:2), (E) BOB@BTFO (1:3) and (F) BOB@BTFO (1:2)-M sample during photocatalytic degradation of RhB.

**
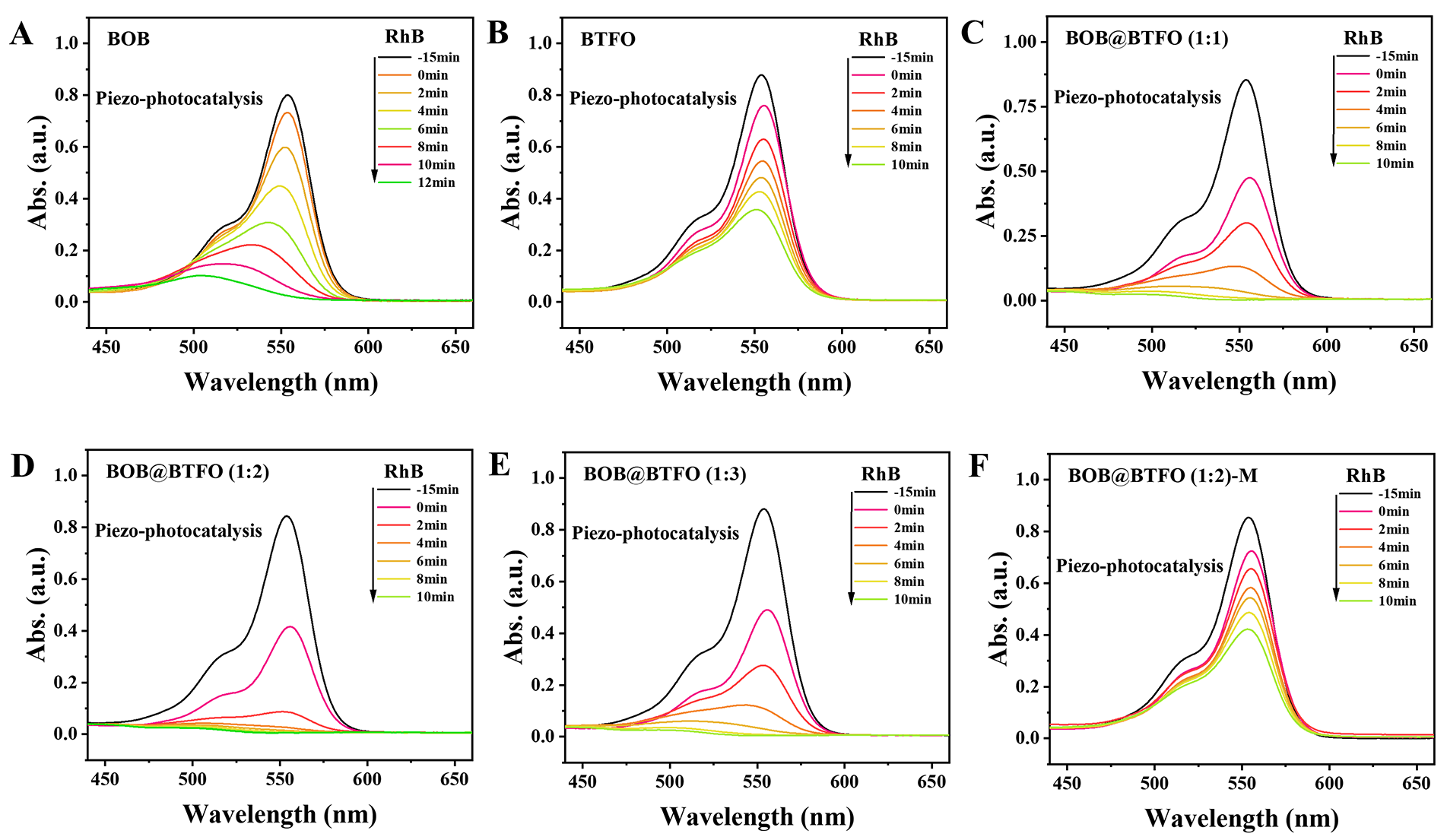
**

**Figure S10.** Absorption spectrum of (A) BOB, (B) BTFO, (C) BOB@BTFO (1:1), (D) BOB@BTFO (1:2), (E) BOB@BTFO (1:3) and (F) BOB@BTFO (1:2)-M sample during piezo-photocatalytic degradation of RhB.


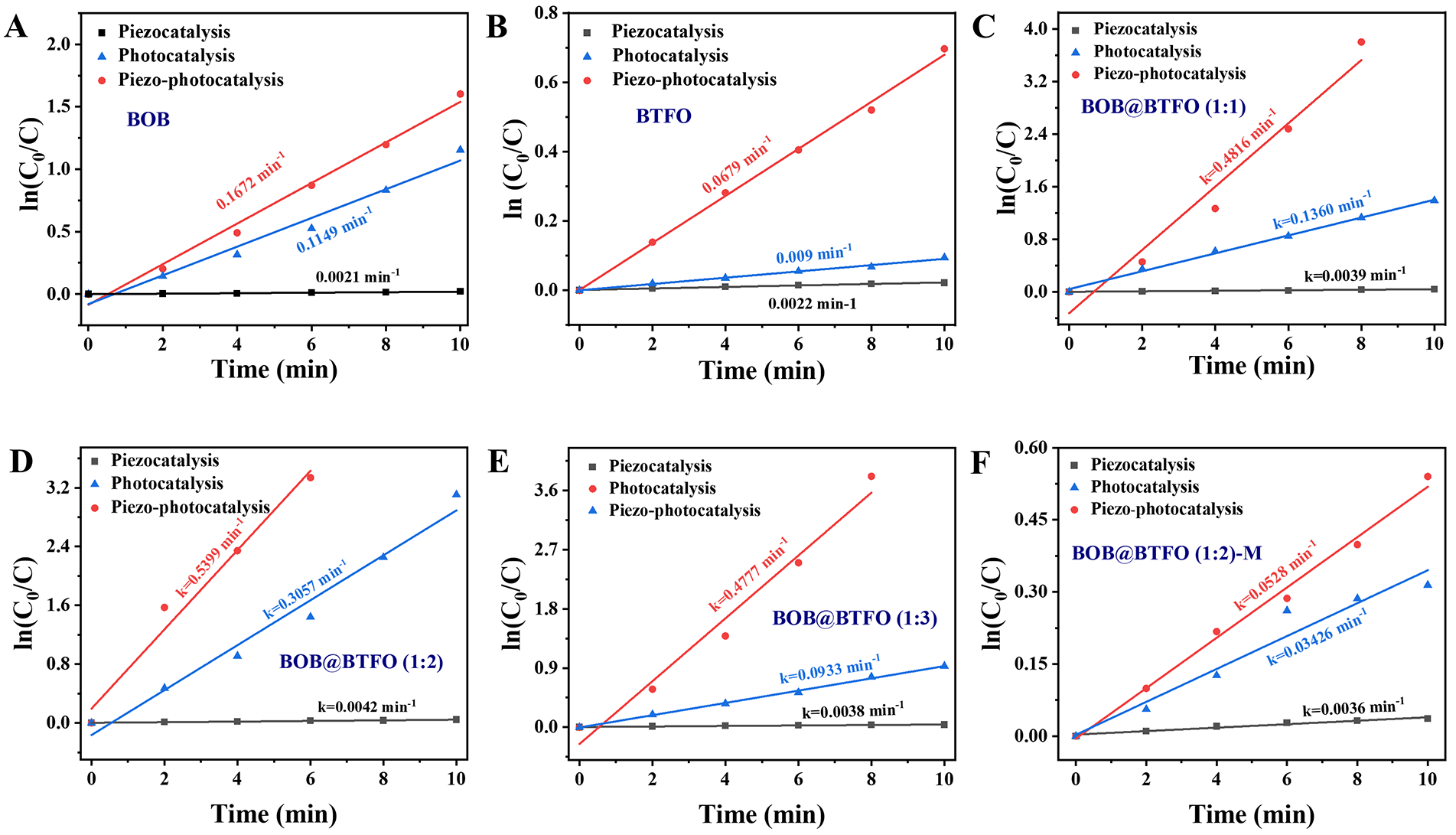


**Figure S11.** First-order fitting of the catalytic performance of (A) BOB, (B) BTFO, (C) BOB@BTFO (1:1), (D) BOB@BTFO (1:2), (E) BOB@BTFO (1:3) and (F) BOB@BTFO (1:2)-M sample during catalytic degradation of RhB.


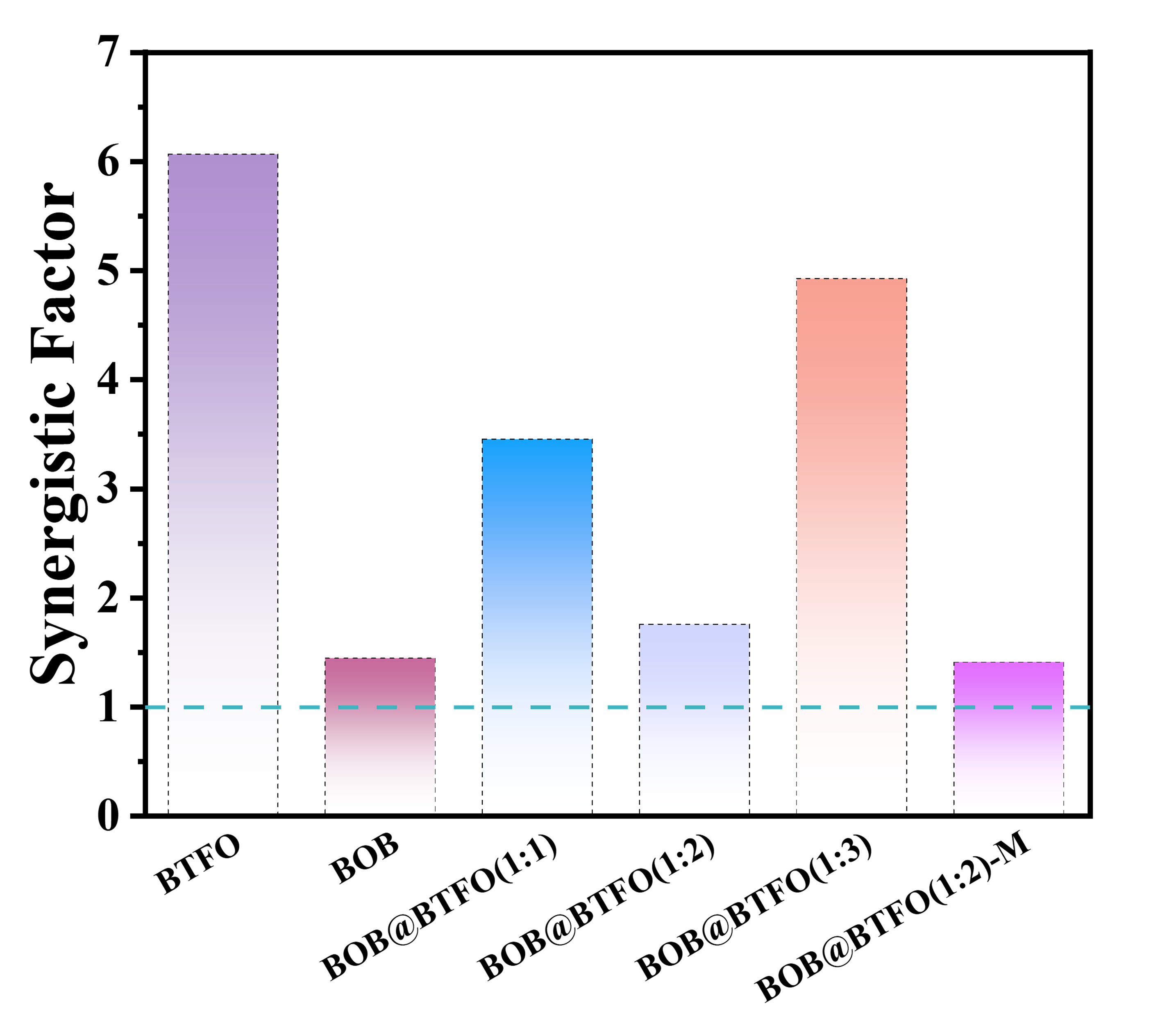


**Figure S12**. The calculated synergistic factor of various materials obtained from the kinetic results.


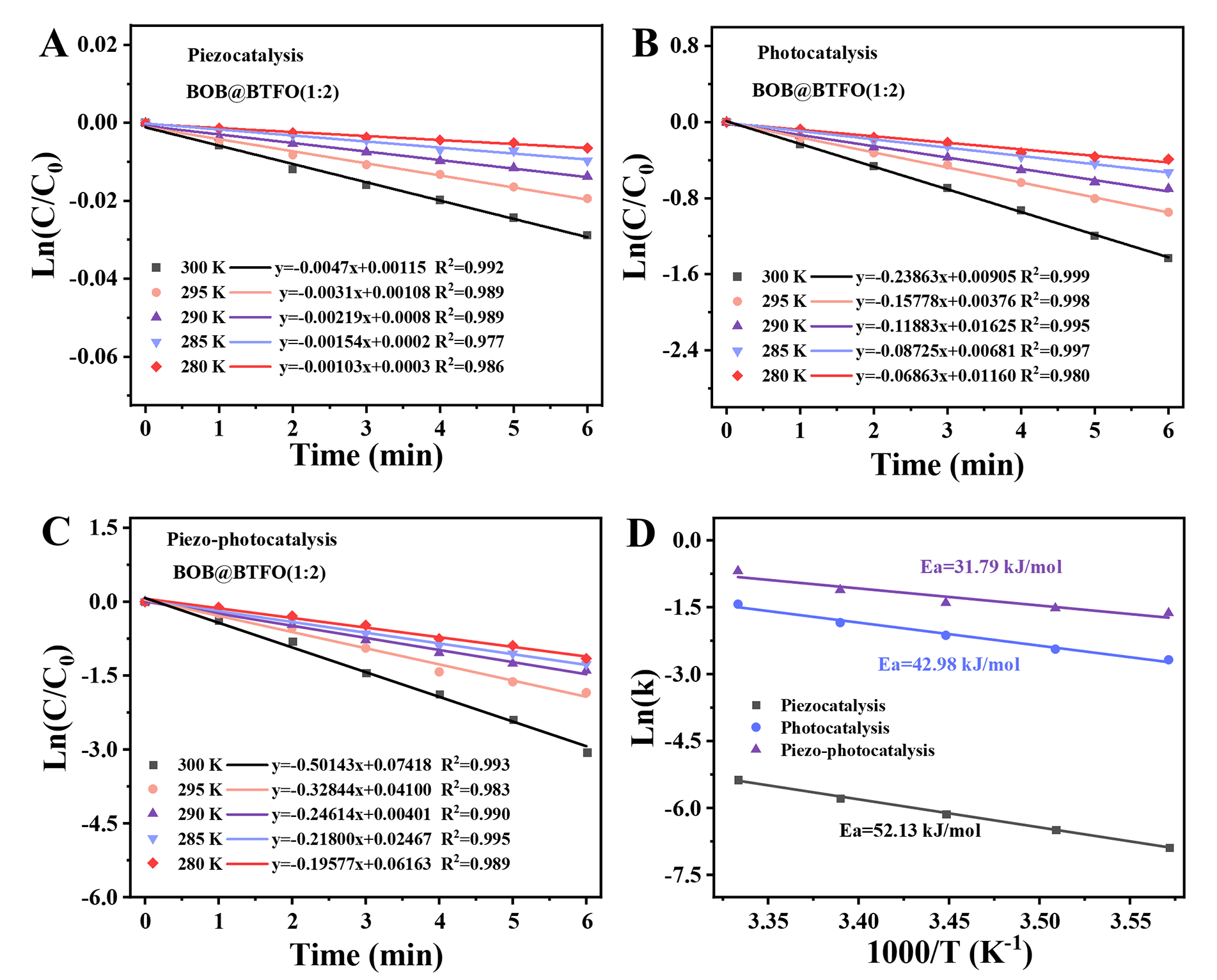


**Figure. S13.** (A-C) The temperature-dependent degradation of RhB using BOB@BTFO (1:2) as a model catalyst. (D) The thermodynamic fittings based on the Eyring analyses.


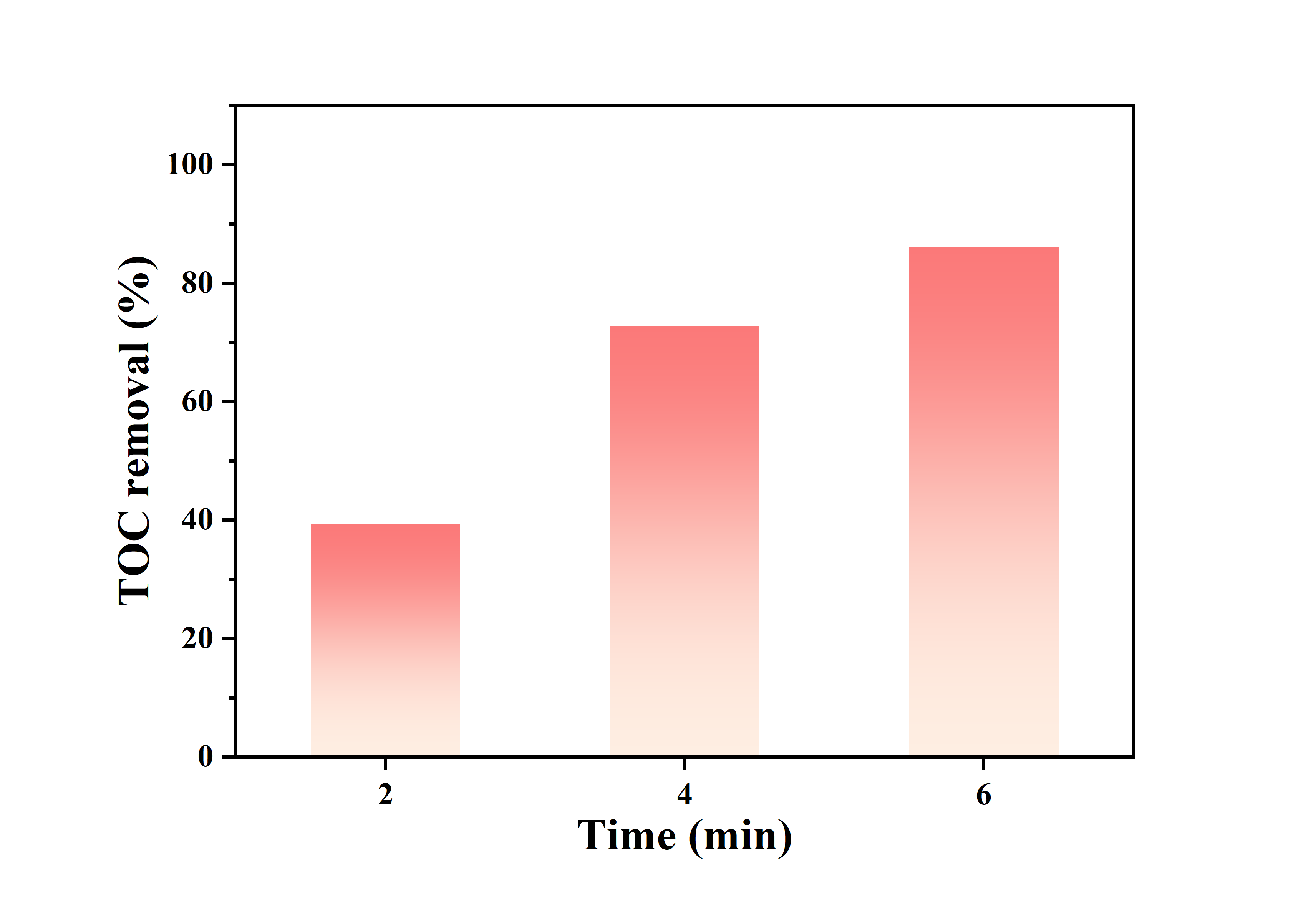


**Figure S14**. TOC removal efficiencies of RhB by BOB@BTFO.


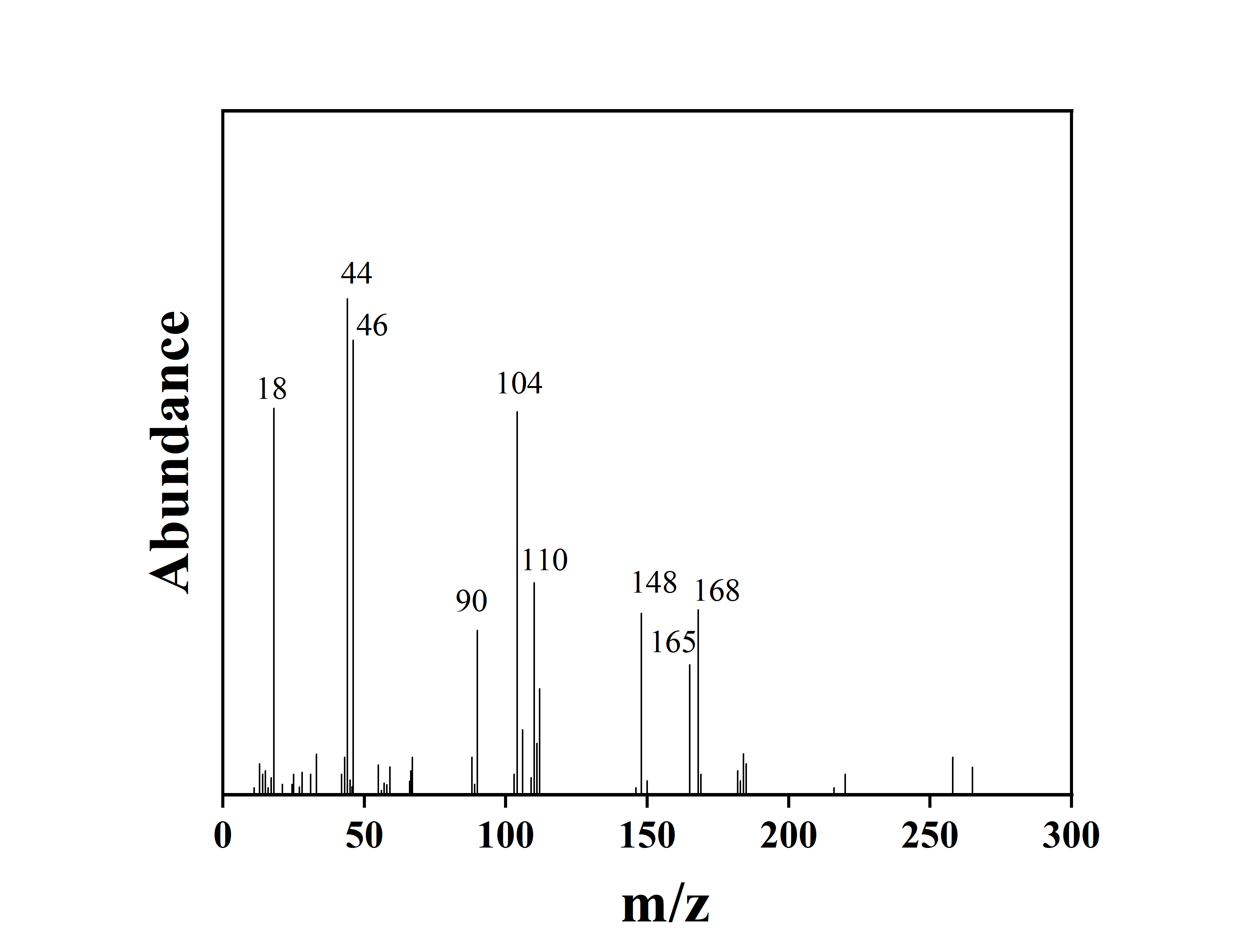


Figure S15. GC/MS results of RhB degradation after 6 min light irradiation.

**Table S2.** Main oxidation products of RhB in the BOB@BTFO system detected during the reaction by GC/MS.

| **m/z** | **Molecular formula** | **Structural** |
| --- | --- | --- |
| 479 | C_28_H_31_N_2_O_3_Cl |  |
| 165 | C_10_H_15_NO |  |
| 168 | C_8_H_8_O_4_ |  |
| 148 | C_9_H_8_O_2_ |  |
| 110 | C_6_H_6_O_2_ |  |
| 104 | C_3_H_4_O_4_ |  |
| 90 | C_2_H_2_O_4_ |  |
| 46 | CH_2_O_2_ |  |
| 44 | CO_2_ |  |
| 18 | H_2_O |  |


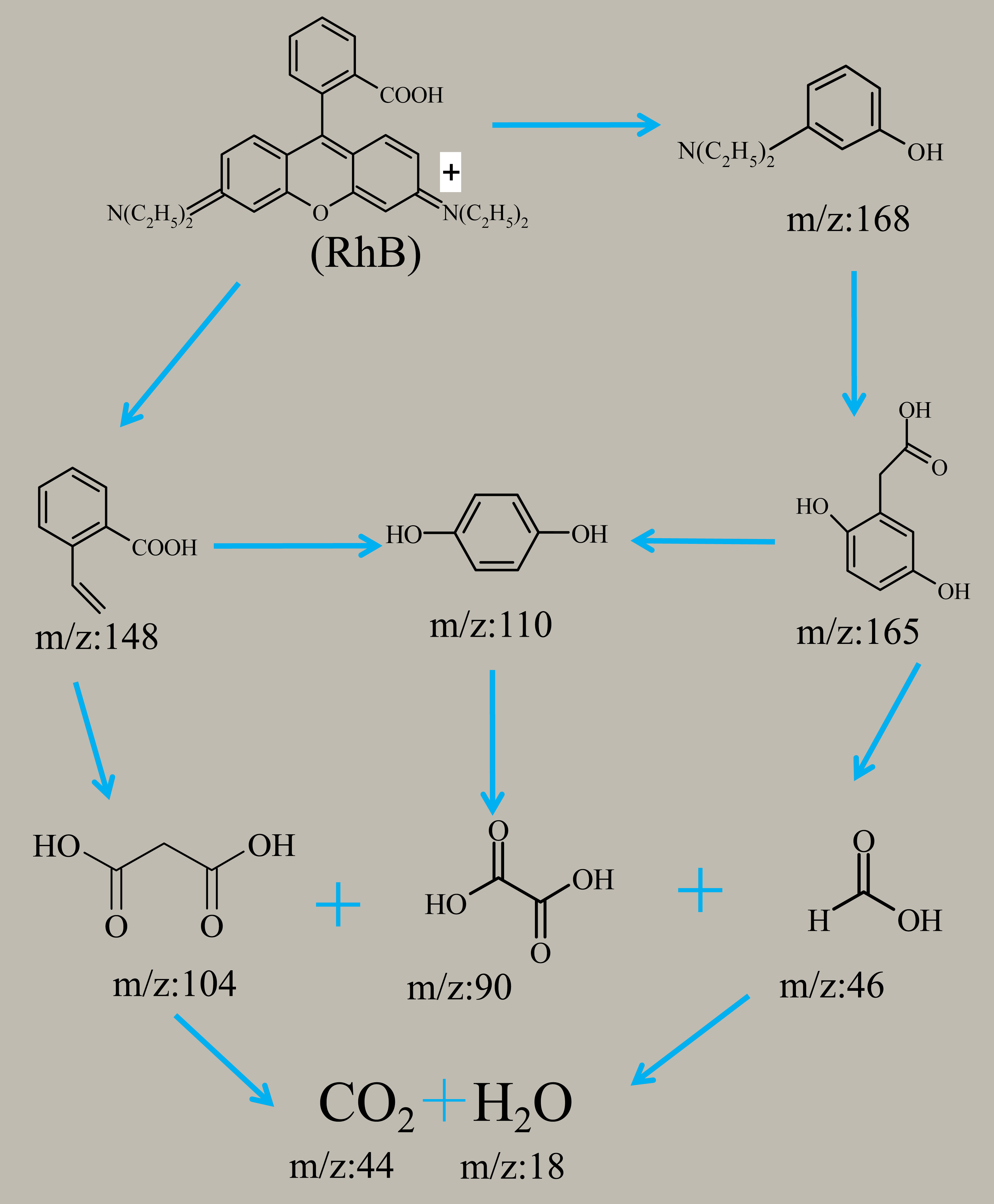


**Figure S16**. Possible degradation pathways of RhB catalyzed by BOB@BTFO.

To further elucidate the potential degradation pathways of Rhodamine B (RhB) by BOB@BTFO, we identified intermediate oxidation products during the reaction using gas chromatography-mass spectrometry (GC-MS). The analytical results, presented in **Figure S15** and **Table S2**, revealed the following degradation intermediates in addition to residual RhB: 3-(diethylamino)phenol (C_10_H_15_NO, m/z 165), 2-(2,5-dihydroxyphenyl)acetic acid (C_8_H_8_O_4_, m/z 168), 2-vinylbenzoic acid (C_9_H_8_O_2_, m/z 148), hydroquinone (C_6_H_6_O_2_, m/z 110), malonic acid (C_3_H_4_O_4_, m/z 104), oxalic acid (C_2_H_2_O_4_, m/z 90), and formic acid (CH_2_O_2_, m/z 46). Based on these intermediates, we propose the degradation pathway illustrated in **Figure S16**. The degradation mechanism initiates with radical species attacking the central carbon atom of RhB, leading to dye decolorization. Subsequent degradation progresses through three primary routes: N-de-ethylation, chromophore cleavage, and aromatic ring opening. These processes progressively break down the complex molecular structure into low molecular weight compounds including hydroquinone, malonic acid, oxalic acid, and formic acid. Ultimately, complete mineralization occurs through the oxidation of these small organic molecules to CO_2_ and H_2_O.


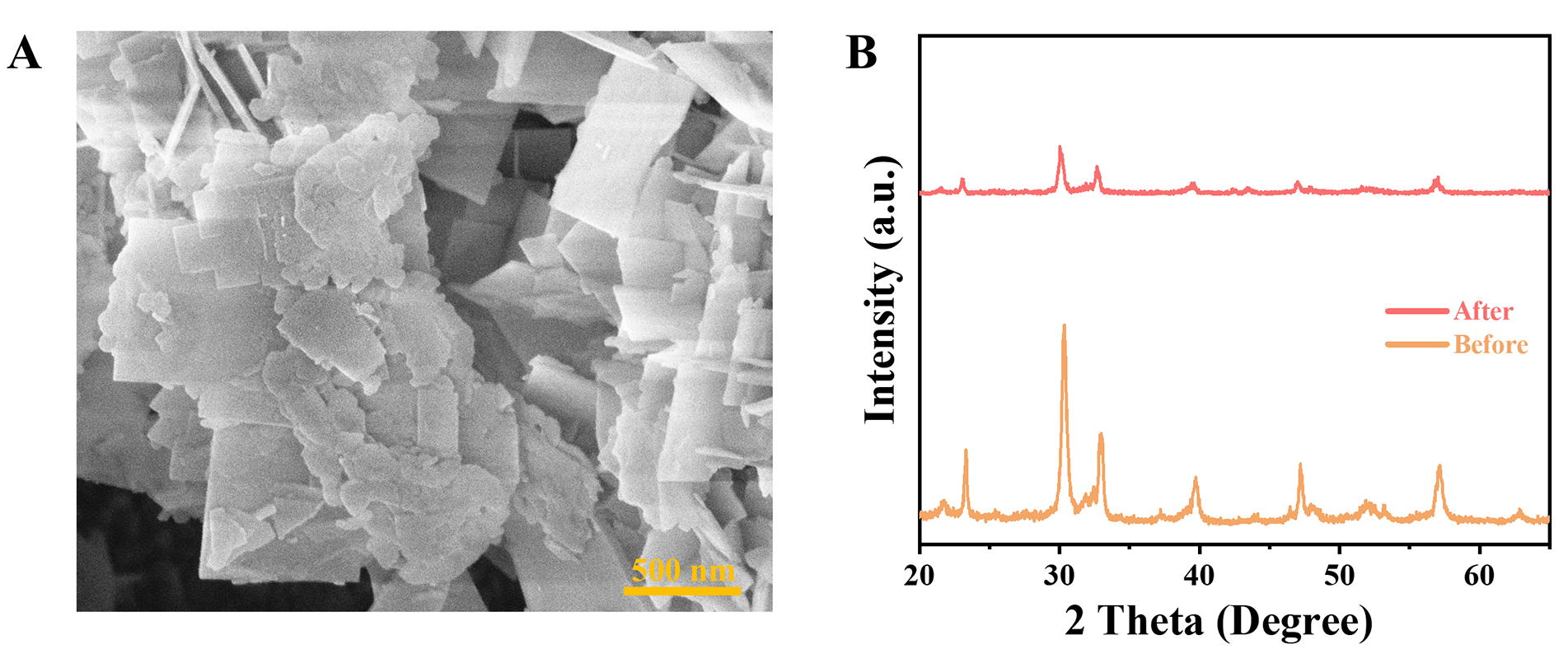


**Figure S17.** (A) SEM and (B) XRD patterns of BOB@BTFO (1:2) before and after reaction.


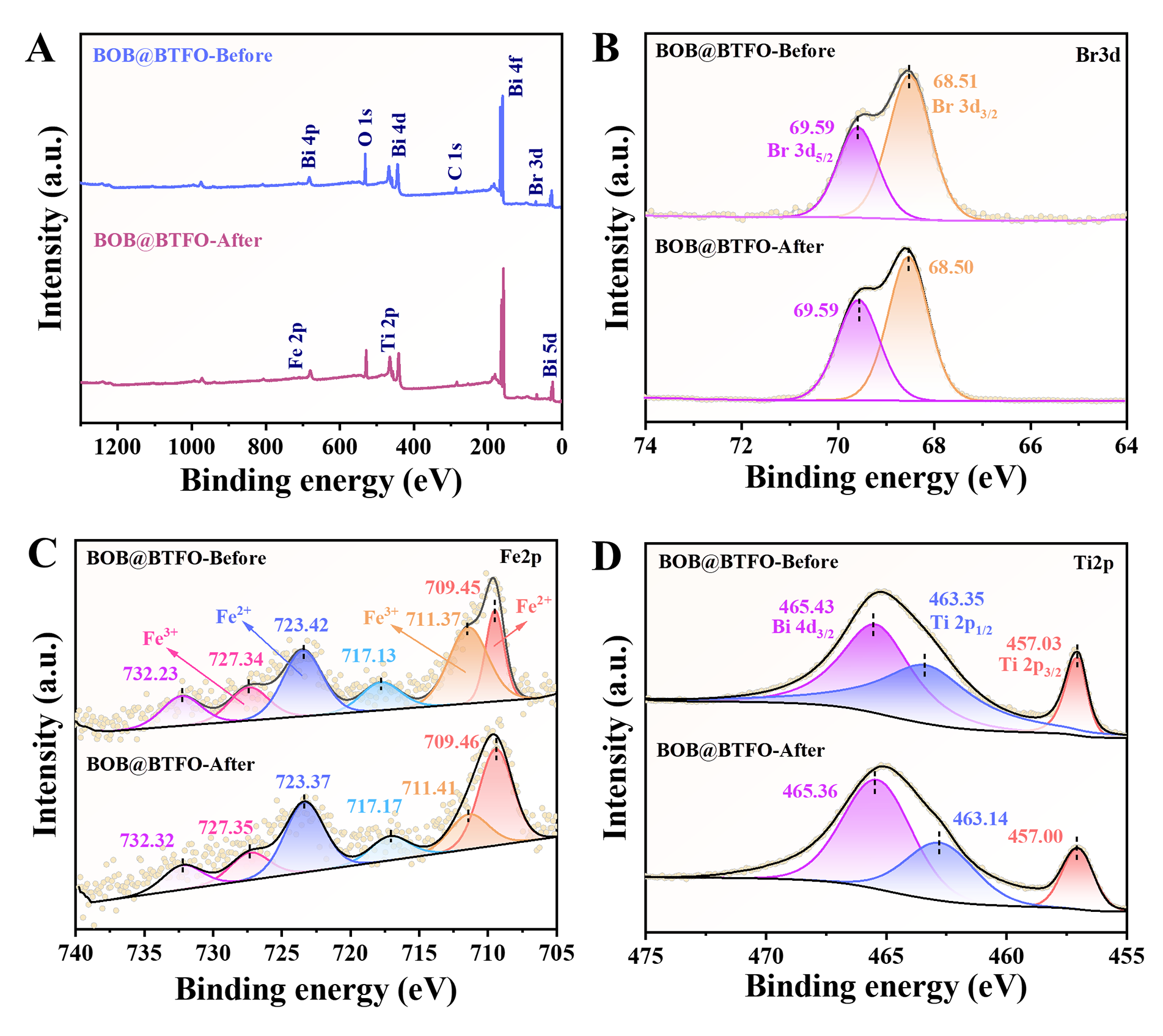


**Figure S18.** (A) XPS full spectra, (B) Br 3d, (C) Fe 2p, (D) Ti 2p of BOB@BTFO (1:2) before and after reaction.

**
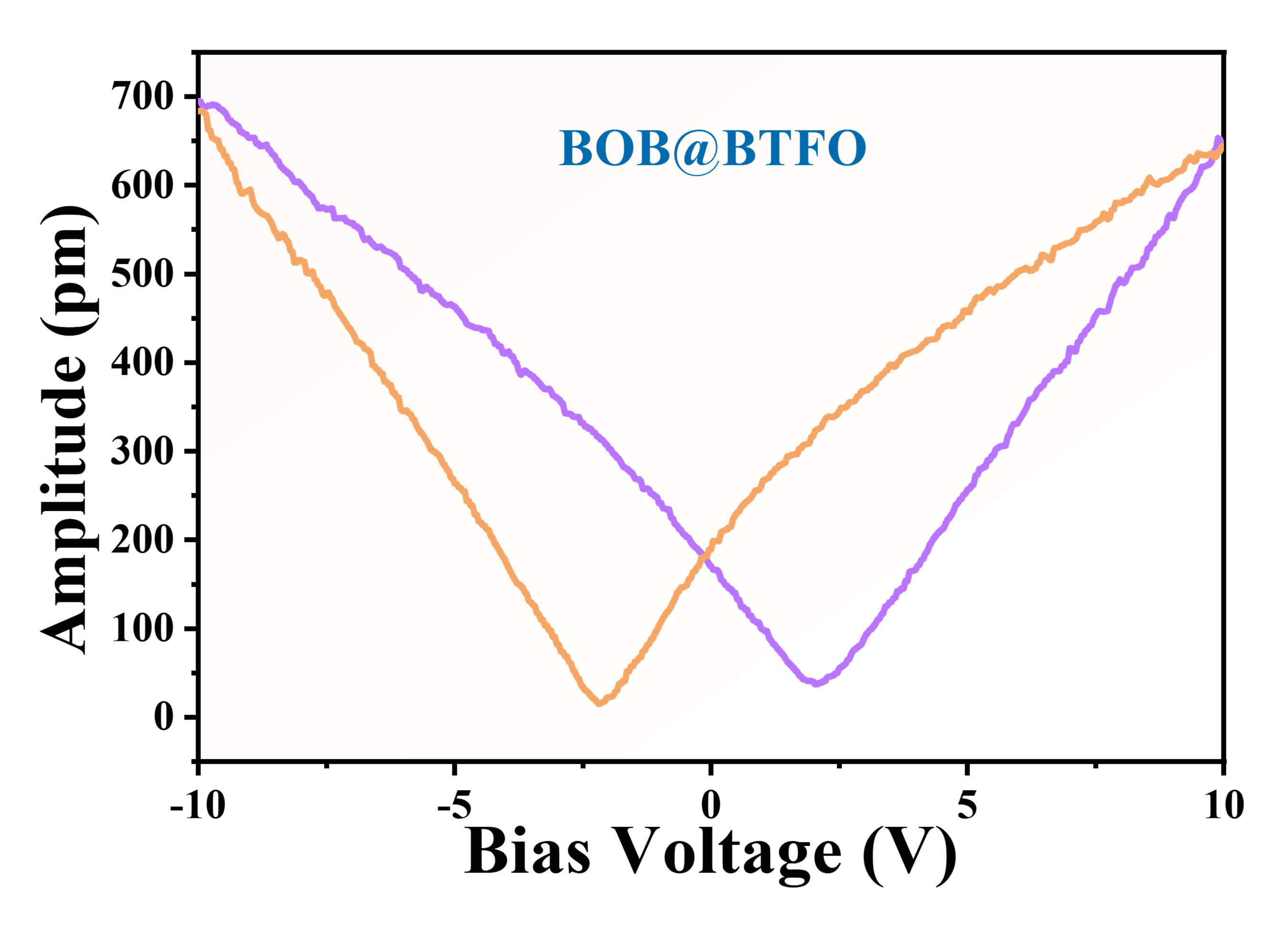
**

**Figure S19.** Piezoelectric amplitude-voltage curves of BOB@BTFO after long-term reaction.


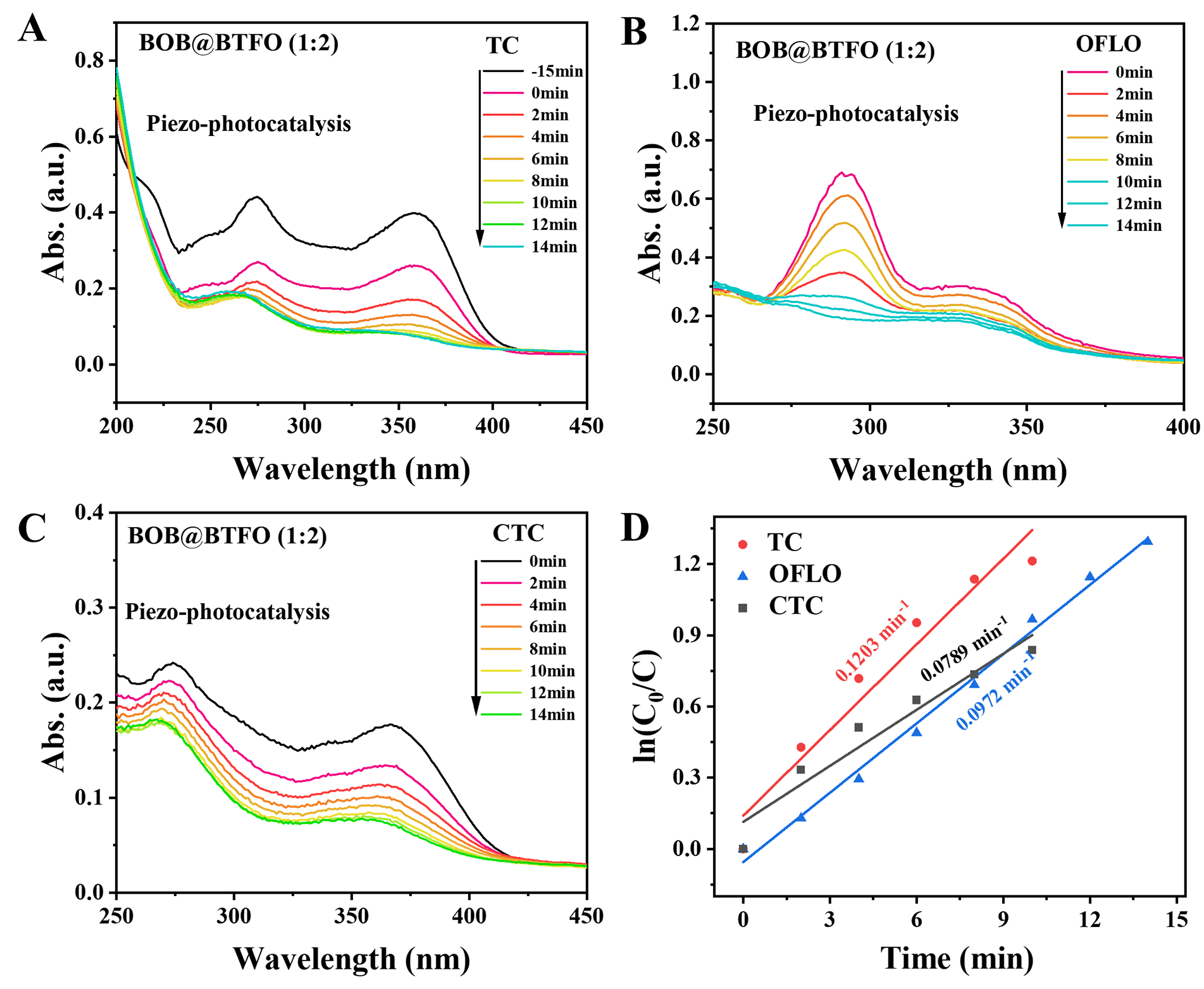


**Figure S20.** Absorption spectrum of BOB@BTFO (1:2) during piezo-photocatalytic degradation of (A) TC, (B) OFLO, (C) CTC. (D) First-order fitting of the catalytic performance of BOB@BTFO (1:2) during catalytic degradation of TC, OFLO and CTC.

**Table S3.** Comparison of the piezo-photocatalytic performance of BOB@BTFO with reported catalyst.

| **Catalysts** | **Degradation target** | **Catalysts dosage (mg/L)** | **Amount**  **of catalyst (mg)** | **Rate constant k (10^-2^ min^−1^)** | **Efficiency** | **Light source**  **/mechanical stimulation** | **Ref.** |
| --- | --- | --- | --- | --- | --- | --- | --- |
| BaTiO_3_ nanoparticles  @TiO_2_ micronflowers | RhB | 10 | 50 | 27.4 | 99.7%  (20 min) | Visible light, ultrasonic | [1] |
| BaTiO_3_ nanosphere  @BiOI nanosheets | RhB | 50 | 100 | 18.76 | 99.7%  (30 min) | Visible light, ultrasonic | [2] |
| BiOBr nanosheets @BaTiO_3_ nanoparticles | RhB | 10 | 30 | 20.839 | 100%  (20 min) | Visible light, ultrasonic | [3] |
| Bi_3_O_4_Cl nanoparticles @Bi_4_O_5_I_2_ nanoparticles | RhB | 10 | 30 | 6.154 | 97.4%  (60 min) | Visible light | [4] |
| Au/Bi_2_WO_6_ microspheres @PVDF films | RhB | 10 | 50 | 6.44 | 98.1%  (60 min) | Visible light, ultrasonic | [5] |
| BiVO_4_ nanoparticles @BiFeO_3_ particles | RhB | 5 | 100 | 3.57 | 98 %  （120 min） | Visible light, ultrasonic | [6] |
| needle-like BaTiO_3_  @ZnO nanofibers | RhB | 5 | 60 | 5.15 | 98.94%  （90 min） | UV-Visible light, ultrasonic | [7] |
| BiFeO_3_ nanoparticles  @BaTiO_3_ nanofibers | RhB | 5 | 50 | 3.8 | 93.20%  （60 min） | UV-Visible light, ultrasonic | [8] |
| MoSe_2_ nanoparticles  @PVDF films | RhB | 20 | 50 | 5.67 | 99.1%  (60 min) | UV-Visible light, Unstirred | [9] |
| Bi_5_Ti_3_FeO_15_  nanofibers | RhB | 5 | 50 | 19.5 | 98%  (20 min) | ultrasonic | [10] |
| Bi_5_Ti_3_FeO_15_  nanoparticles | RhB | 5 | 50 | 6.1 | 94%  (50 min) | ultrasonic | [11] |
| BiOBr nanosheets @Bi_5_Ti_3_FeO_15_  nanosheets | RhB | 25 | 50 | 53.99 | 100%  (6 min) | UV-Visible light, ultrasonic | This work |
| Bi_4_Ti_3_O_12_ nanosheets  @BiOCl microflowers | TC | 10 | 50 | 10.80 | 80.41%  (15 min) | UV-Visible light, ultrasonic | [12] |
| BiOBr nanosheets @Bi_5_Ti_3_FeO_15_  nanosheets | TC | 10 | 50 | 12.03 | 78.70%  (12 min) | UV-Visible light, ultrasonic | This  work |
| BP+BiVO_4_ microtubes | CTC | 10 | 150 | 1.6 | 88%  (120 min) | Visible light，ultrasonic | [13] |
| ZnIn_2_S_4_ microsphere  @GaN thin film | CTC | 20 | 5 | 0.503 | 85%  (320 min) | Visible light | [14] |
| BiOBr nanosheets @Bi_5_Ti_3_FeO_15_  nanosheets | CTC | 10 | 50 | 7.89 | 69.1%  (14 min) | UV-Visible light, ultrasonic | This  work |
| g-C_3_N_4_ nanosheets @Ag_3_PO_4_ nanoparticles | OFLO | 10 | 250 | 9.29 | 74.6%  (30 min) | Visible light | [15] |
| BiOBr nanosheets @Bi_5_Ti_3_FeO_15_  nanosheets | OFLO | 10 | 50 | 9.72 | 80.20%  (20 min) | UV-Visible light, ultrasonic | This  work |


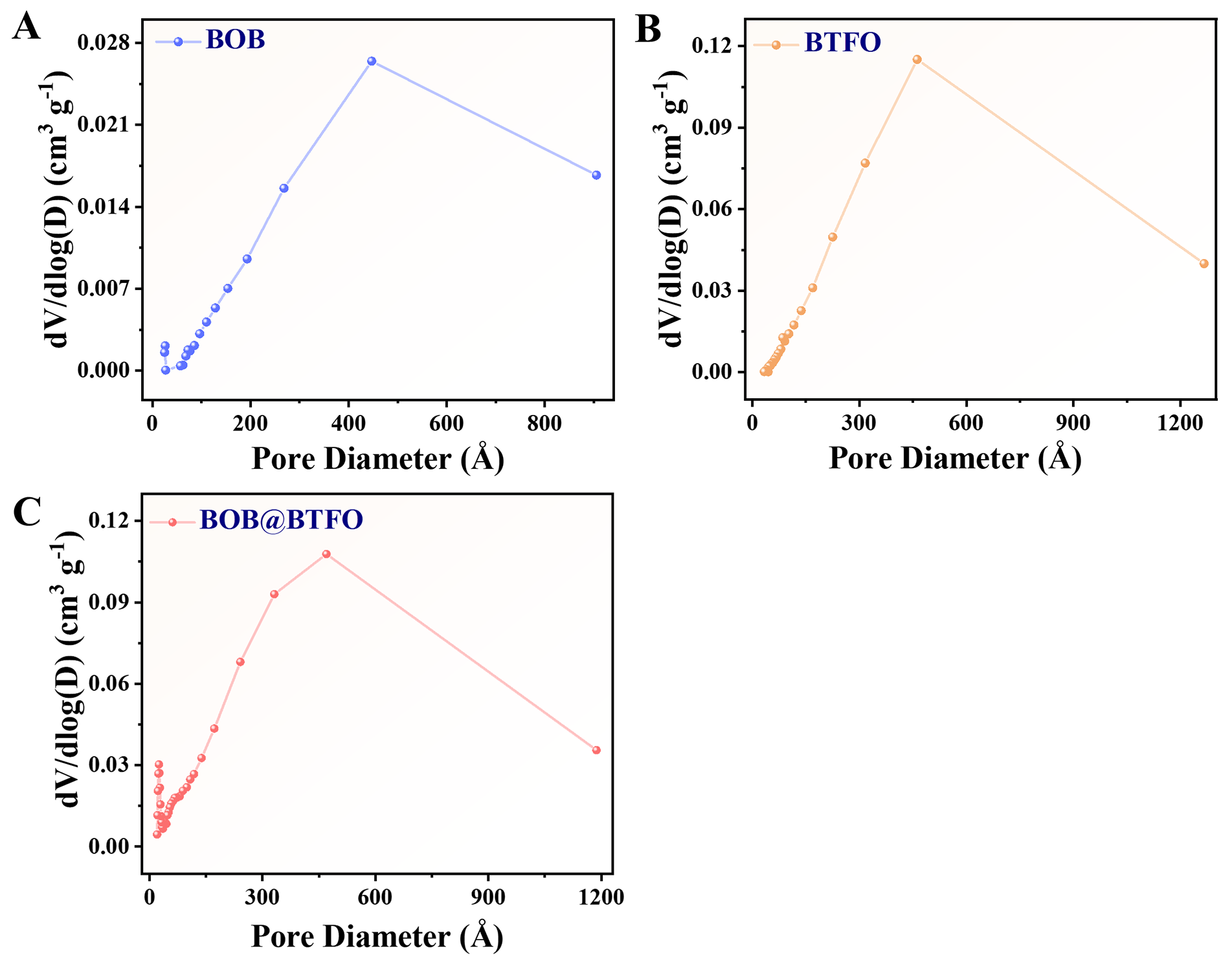


**Figure S21.** Corresponding pore size distribution curve of (A) BOB, (B) BTFO, (C) BOB@BTFO.


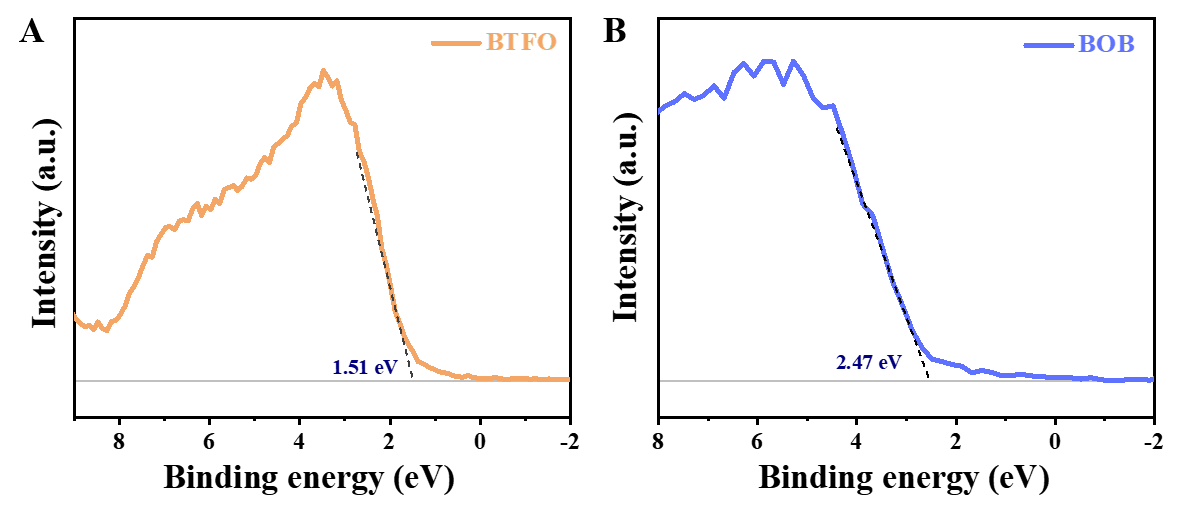


**Figure S22.** Valence band XPS spectra of (A) BTFO and (B) BOB.


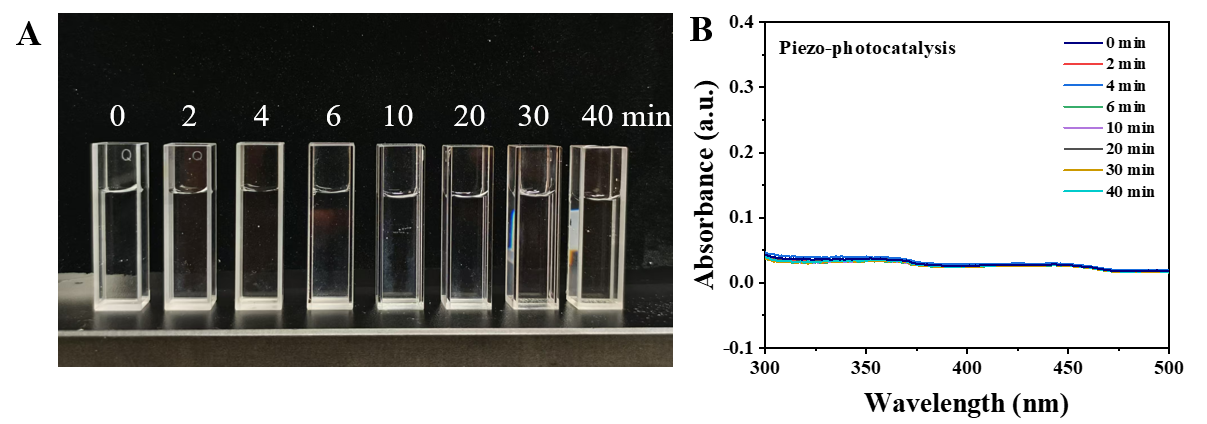


**Figure S23**. (A) Photograph of aqueous solution by KI colorimetric method in the piezo-photocatalytic process from 0 to 40 min using BOB@BTFO. (B) The absorbance and yield of generated H_2_O_2_ in the piezo-photocatalytic process by BOB@BTFO.


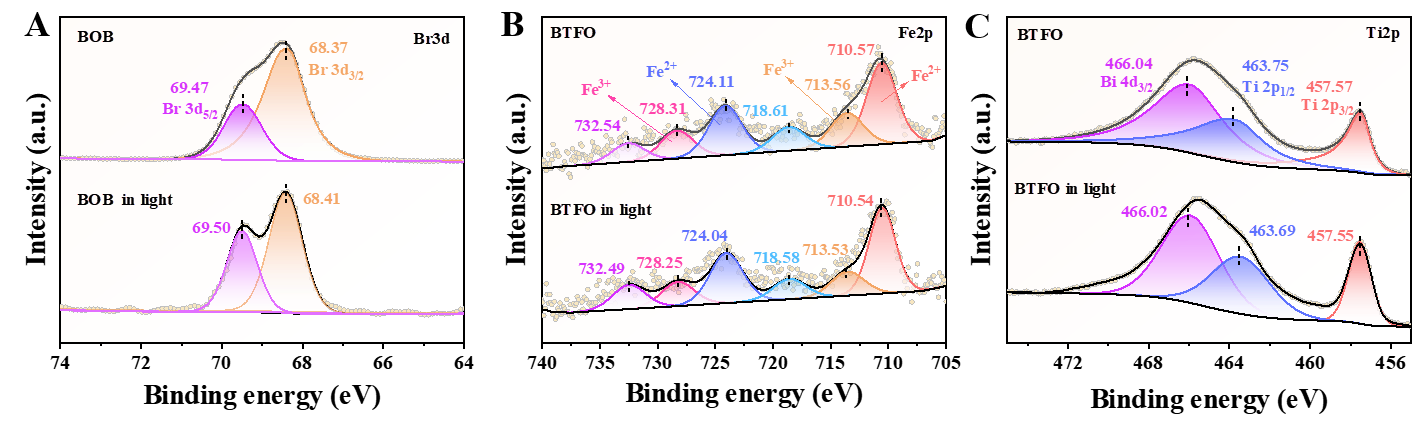


**Figure S24.** (A) High-resolution XPS Br 3d of BOB, (B) Fe 2p, (C) Ti 2p of BTFO with and without light irradiation.


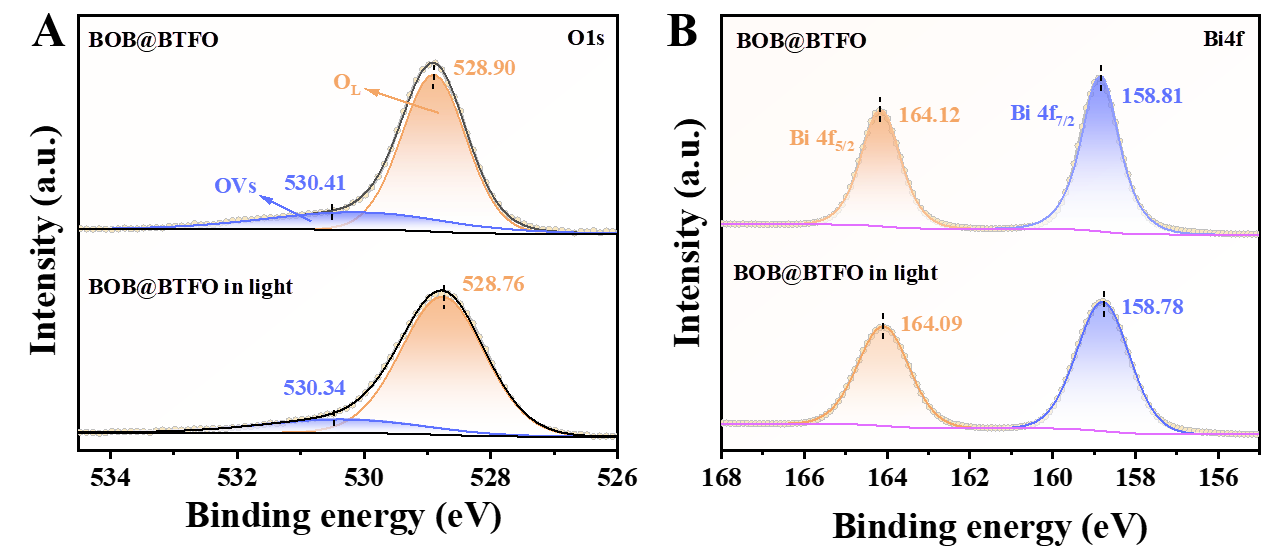


**Figure S25.** High-resolution XPS of BOB@BTFO: (A) O 1s, (B) Bi 4f with and without light irradiation.


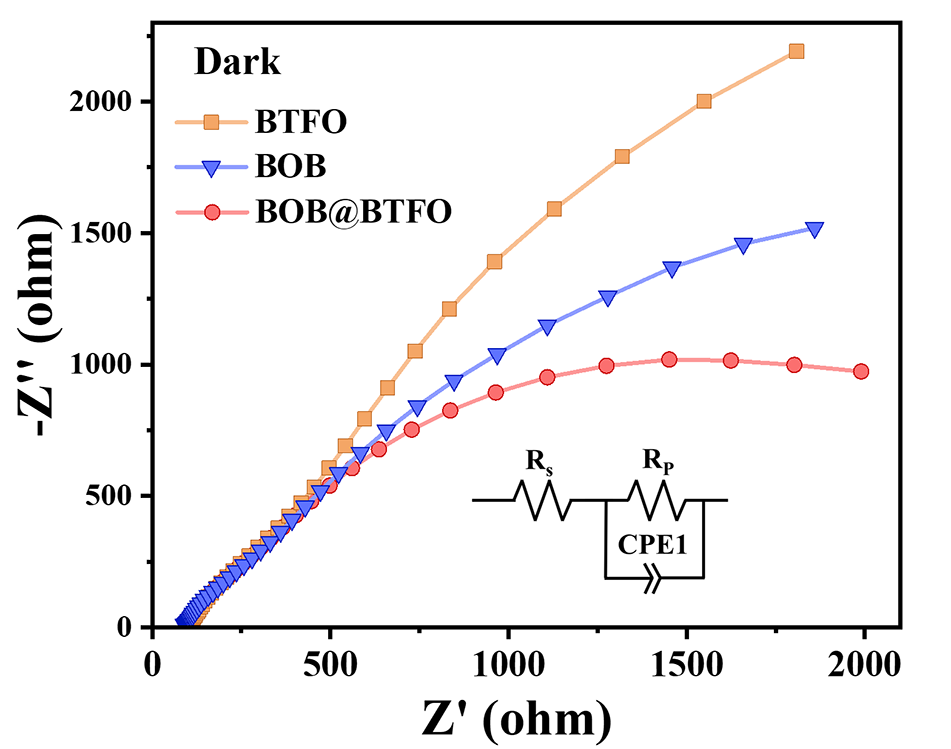


**Figure S26.** The EIS Nyquist plots of BOB, BTFO and BOB@BTFO in dark.

**Table S4.** Impedance fitting parameters for BOB, BTFO and BOB@BTFO.

| **Catalysts** | **Rs** **(****Ω)** | **Rp (Ω)** | **CPE-T**  **(Ω^-1^ cm^-2^ s^n^)** | **CPE-P**  **(Ω^-1^ cm^-2^ s^n^)** |
| --- | --- | --- | --- | --- |
| BOB | 79.9 | 1098 | 3.0175×10^-4^ | 0.68519 |
| BTFO | 93.04 | 20011 | 1.2895×10^-4^ | 0.64461 |
| BOB@BTFO | 86.65 | 369 | 3.2830×10^-4^ | 0.58092 |


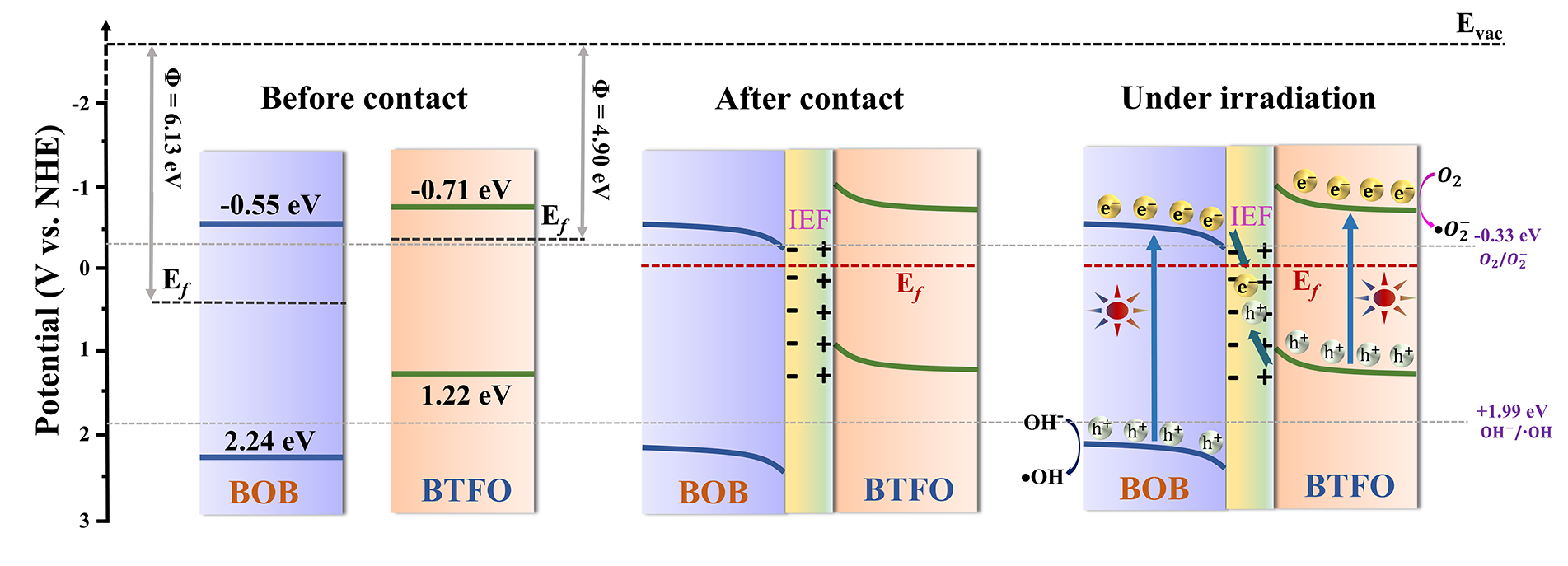


**Figure S27.** Schematic illustration of BOB and BTFO band structure in before contact, after contact, and under irradiation.

**References**

[1] Q. Liu, F. Zhan, H. Luo, D. Zhai, Z. Xiao, Q. Sun, Q. Yi, Y. Yang, D. Zhang, *Appl. Catal. B Environ.* **2022**, *318*, 121817.

[2] G. Liu, H. Fei, Z. Feng, Q. Shao, T. Zhao, W. Guo, F. Li, *J. Clean. Prod.* **2024**, *440*, 140886.

[3] S. Zhong, Y. Wang, Y. Chen, X. Jiang, M. Lin, C. Lin, T. Lin, M. Gao, C. Zhao, X. Wu, *Chem. Eng. J.* **2024**, *488*, 151002.

[4] Y. Li, S. Liu, L. Huang, S. Shu, J. Yao, M. Zhu, Y. Li, L. Qiu, L. Huang, S. Fu, *J. Colloid Interface Sci.* **2023**, *652*, 798.

[5] T. Song, G. Li, X. Yu, J. Xia, Q. Deng, X. Liu, Y. Gao, *Appl. Surf. Sci.* **2025**, *679*, 161163.

[6] Q. Jing, Z. Liu, X. Cheng, C. Li, P. Ren, K. Guo, H. Yue, B. Xie, T. Li, Z. Wang, others, *Chem. Eng. J.* **2023**, *464*, 142617.

[7] W. Zheng, Y. Tang, Z. Liu, G. Xing, K. Zhao, *J. Mater. Chem. A* **2022**, *10*, 13544.

[8] M. Li, J. Zhou, R. Di, Z. Zhang, X. Mu, X. Wang, Y. Gu, L. Su, J. Liu, C. Liu, *J. Adv. Ceram.* **2024**, *13*, 2030.

[9] X. Chen, A. Li, L. Xing, J. Wang, Y. Sun, Y. Wang, G. Chen, T. Xing, L. Xu, *J. Water Process Eng.* **2024**, *59*, 105015.

[10] X. Zhu, X. Wu, Y. Li, W. Shao, J. Fu, Q. Lin, J. Tan, S. Gao, Y. Zhang, W. Ye, *ACS Appl. Nano Mater.* **2023**, *6*, 5602.

[11] Y. Zheng, X. Wu, Y. Zhang, Y. Li, W. Shao, J. Fu, Q. Lin, J. Tan, S. Gao, W. Ye, *Chem. Eng. J.* **2023**, *453*, 139919.

[12] T. Liu, Y. Li, Z. Zheng, P. Jia, Y. Wang, *J. Alloys Compd.* **2023**, *966*, 171572.

[13] M. Zhang, Q. Shi, X. Cheng, J. Yang, Z. Liu, T. Chen, Y. Qu, J. Wang, M. Xie, W. Han, *Chem. Eng. J.* **2020**, *400*, 125871.

[14] Y. He, J. Shi, Q. Yang, Y. Tong, Z. Ma, L. B. Junior, B. Yao, *Chem. Eng. J.* **2022**, *446*, 137355.

[15] R. Chen, S. Ding, N. Fu, X. Ren, *J. Environ. Chem. Eng.* **2023**, *11*, 109440.

[16] Y. Wang, H. Ma, J. Liu, Z. Zhang, Y. Yu, S. Zuo, *J. Colloid Interface Sci.* **2024**, *665*, 655.

[17] Q. Pan, J. Wang, H. Chen, P. Yin, Q. Cheng, Z. Xiao, Y. Zhao, H.-B. Liu, *J. Water Process Eng.* **2023**, *56*, 104330.
